# Rare Copy Number Variants Identified Suggest the Regulating Pathways in Hypertension-Related Left Ventricular Hypertrophy

**DOI:** 10.1371/journal.pone.0148755

**Published:** 2016-03-01

**Authors:** Hoh Boon-Peng, Julia Ashazila Mat Jusoh, Christian R. Marshall, Fadhlina Majid, Norlaila Danuri, Fashieha Basir, Bhooma Thiruvahindrapuram, Stephen W. Scherer, Khalid Yusoff

**Affiliations:** 1 Institute of Medical Molecular Biotechnology, Faculty of Medicine, Universiti Teknologi MARA, Sungai Buloh Campus, Jalan Hospital, 47000 Sungai Buloh, Selangor, Malaysia; 2 UCSI University, Jalan Menara Gading, UCSI Heights, 56000 Kuala Lumpur, Wilayah Persekutuan Kuala Lumpur, Malaysia; 3 The Centre for Applied Genomics, The Hospital for Sick Children, Toronto, Ontario, Canada; 4 McLaughlin Centre and Department of Molecular Genetics, University of Toronto, Toronto, Canada; 5 Faculty of Medicine, Universiti Teknologi MARA, Sungai Buloh Campus, Jalan Hospital, 47000 Sungai Buloh, Selangor, Malaysia; Osaka University Graduate School of Medicine, JAPAN

## Abstract

Left ventricular hypertrophy (LVH) is an independent risk factor for cardiovascular morbidity and mortality, and a powerful predictor of adverse cardiovascular outcomes in the hypertensive patients. It has complex multifactorial and polygenic basis for its pathogenesis. We hypothesized that rare copy number variants (CNVs) contribute to the LVH pathogenesis in hypertensive patients. Copy number variants (CNV) were identified in 258 hypertensive patients, 95 of whom had LVH, after genotyping with a high resolution SNP array. Following stringent filtering criteria, we identified 208 rare, or private CNVs that were only present in our patients with hypertension related LVH. Preliminary findings from Gene Ontology and pathway analysis of this study confirmed the involvement of the genes known to be functionally involved in cardiac development and phenotypes, in line with previously reported transcriptomic studies. Network enrichment analyses suggested that the gene-set was, directly or indirectly, involved in the transcription factors regulating the “foetal cardiac gene programme” which triggered the hypertrophic cascade, confirming previous reports. These findings suggest that multiple, individually rare copy number variants altering genes may contribute to the pathogenesis of hypertension-related LVH. In summary, we have provided further supporting evidence that rare CNV could potentially impact this common and complex disease susceptibility with lower heritability.

## Introduction

Cardiovascular diseases remain the most significant cause of mortality globally, in high- and middle- or low-income countries [1]. Hypertension is the main driver for this epidemiologic reality [2,3]. Left ventricular hypertrophy (LVH) is a common outcome of hypertension, especially when uncontrolled whereby the LV wall thickens and/or LV mass increases in response to the biomechanical stress rendered by the elevated blood pressure, which is initially compensatory to the wall stress [4–6]. Hypertension related LVH is a complex, multifactorial and polygenic pathophysiologic condition. Almost a third of hypertensive patients develop LVH, [7], despite controlled with anti-hypertensive medications in more than half of them [8]. Thus while elevated blood pressure may explain the development of LVH, there may be other contributory factors. Hereditary may contribute up to 60% of this risk for developing LVH [9,10]

Although LVH can be reversed by pharmacological control of blood pressure such as losartan, identifying those at risk of developing LVH may have significant impact on the prognosis of patients with hypertension by providing a means of prevention of its development. Hence identifying the causative genes and / or the core biological pathway(s) leading to pathogenesis of LVH in hypertension is crucial in addressing such question.

At present, the fundamental hypothesis for genetic influence on complex diseases predominantly lies on the “common disease–common variant (CD-CV)” model [11,12] in which a disease trait is caused by a combination of common alleles (defined as ≥ 5% in a population), each contributing modest additive effects. Although several Genome Wide Association (GWA) studies have been conducted, only a handful of SNPs associated with left ventricular hypertrophy (LVH) have been identified [13,14] (https://www.genome.gov/). A number of genes known to play a role in the susceptibility of hypertension related-LVH such as angiotensin converting enzyme have not been able to be detected [15–17]. While another major candidate gene calcineurin has recently been reported in animal model [18], but it was not found in GWA studies for human. Recently an alternative hypothesis has been proposed to explain the failure to detect associations, namely the “missing heritability”, [11] where rarer variants are believed to carry a relatively larger effect on complex disease susceptibility. In an attempt to map the susceptible genes of hypertensive LVH, we adopted an alternative approach, with a postulation that some variants predisposing to hypertension related LVH are highly penetrant, individually rare or population specific, and of recent origin, even specific to single case [19,20].

## Materials and Methods

### Sample recruitment

A total of 116 blood samples of the hypertensive subjects were recruited from the PURE (Prospective Urban-Rural Epidemiologic) / REDISCOVER (REsponDing to IncreaSing CardiOVascular disEase pRevalence) Study from 2007 to 2010 carried out in Malaysia [21]. We defined the control group as those hypertensive patients without LVH; while cases were defined as those hypertensive patients with LVH. The following inclusion criteria were used for sample recruitment:

1. 30–60 years of age
2. Hypertension defined as systolic blood pressure ≥140 mmHg and/or diastolic blood pressure ≥90 mmHg

The exclusion criteria of the study were:

1. Prescribed anti-hypertensive drugs at the time of enrolment
2. Smoker
3. Alcohol drinkers
4. Diabetes

### Echocardiography

Echocardiographic measurements were made using the Echo Pac in the Non Invasive Cardiac Lab, Universiti Teknologi MARA, Faculty of Medicine Selayang Campus. Doppler, two-dimensional (2D), and M-mode (2D-guided, in the parasternal short axis view) echocardiograms were performed using a standard protocol. Measurements were made using a computerized review station equipped with a digitizing tablet and monitor overlay for calibration and quantification.

Transthorasic echocardiogram criteria for LV mass index used the formula: LV mass index = (0.8 (1:04 ([LVIDD PWTD IVSTD] ^3^—[LVIDD] ^3^) (0.6g / Height^2^) (Devereux Criteria), where:

LVIDD = Left ventricular internal dimension in diastole
PWTD = Posterior wall thickness at end in diastole
IVSTD = Interventricular septal thickness at end-diastole

Subjects were diagnosed as LVH when Left Ventricular Mass Index (LVMI) exceeded 110 g/m^2^ in women and 125 g/m^2^ in men.

All subjects provided written informed consent. Ethics approval was obtained from ethics committees of the Universiti Teknologi MARA (UiTM)[REC/UITM/2007(10)].

### Microarray analysis

Genomic DNA was extracted either from whole, or clotted blood using commercially available kit. Genotyping was carried out with the Illumina Human 660W-Quad Beadchip (San Diego, SA, USA). Briefly, 500 ng of genomic DNA was denatured overnight, and enzymatically fragmented, precipitated with isopropanol, centrifuged at 4°C, and resuspended in hybridization buffer. All beadchips were prepared for hybridization in a capillary flow-through chamber. Samples were loaded to beadchips and incubated overnight in the Illumina Hybridization Oven. Unhybridized or non-specific products were washed, and beadchips were preceded with staining and extension. The beadchips were scanned on the Illumina Beadarray Reader using default settings, and intra-chip normalization was performed using Illumina Genome Studio with a GenCall cut-off point 0.1 and call rare cut-off of 99%. Built-in controls–both sample dependent and sample independent, were inspected to assess the quality of the experiment.

### CNV detection, quality control and analysis of rare CNV

The Log R ratio (LRR) and B allele frequencies (BAF) were first exported from Genome Studio (Illumina). The Illumina cluster file comprising >120 HapMap samples was used as reference to generate intensities and genotypes. Stringent criteria of quality control were applied to the array [22–24]. Samples were excluded if: (i) genotype call rate of <99%; (ii) LRR values with a standard deviation above 0.35; (iii) standard deviation for B allele frequencies of >0.13; (iv) cross samples batch normalized ratio standard deviation >0.27.

Samples passing QC were carried out for further analysis. CNVs were called using three independent algorithms: CNV partition v2.3.4 (Genome Studio, Illumina), PennCNV (Wang et al., 2007) and iPattern (The Centre for Applied Genomics, Toronto). The application of multiple algorithms minimizes the number of potential false positive discoveries; and thus increases the chance of obtaining more high confidence calls [24].

CNV analyses were performed using the original array coordinates based on Human Genome Assembly NCBI (Build hg18). We applied stringent filtering criteria in CNV analysis by excluding CNVs calls with: (i) less than five consecutive probes; (ii) located in regions with high GC content (>70%); (iii) approximately 30 kb adjacent to the centromere or telomeres; (iv) size less than 1 Kb; (iv) sex chromosomes; and (v) called by only one out of the three algorithms.

In addition, manual visual inspection was used to exclude potential false positives (typically >1 Mb) due to unknown artefacts. We excluded samples outliers with respect to executive aggregate length of CNVs.

CNVs that passed all QCs were considered rare or novel if they: (i) did not overlap with any known copy number polymorphism (frequency >1%); (ii) had <50% reciprocal overlap (by length) with CNVs reported in Database for Genomic Variants (DGV), whereby the ‘case’ CNV overlapped with at least 50% of the control CNV and the conversely, the control CNV overlapped with at least 50% of the case CNV [23]; (iii) occurred as singleton in the 116 samples genotyped in this study. In other word, this means the rare CNVs in this study are uniquely identified for both length and locus, via the comparison to known variants with less than 50% overlap. Recurrent CNVs specific to case group in this study were also identified.

The rare and/or recurrent CNVs specific to the case group identified in this study were further assessed by comparing to control sample datasets from HapMap3 and subsequently from Singapore Genome Variation Project (SGVP, http://www.statgen.nus.edu.sg/~SGVP/) as the population matched controls. CNVs in cases with >50% reciprocal overlap to these control datasets were excluded. We limited our rare CNVs cut-off size of >1 kb, instead of >30 kb suggested by most authors [19,20,24].

### Replication study

We replicated the study on additional 143 samples, consisting 51 case and 92 controls. CNV typing was carried out using Illumina OmniExpress (San Diego, SA, USA), comprising >750,000 SNV probes according to the manufacturer’s protocols. Criteria for CNV calls were as mentioned above. The CNVs were called using PennCNV, QuantiSNP [25] and iPattern.

### Pathway analysis

Gene Ontology (GO) analysis was carried out using, the DAVID (Database for Annotation, Visualization and Integrated Discovery, version 6.7) (david.abcc.ncifcrf.gov), Ingenuity (http://www.ingenuity.com/) and GeneGO Metacore (https://portal.genego.com/). An interaction network was generated on the”case-specific” genes, using MetaCore (GeneGo).

### qPCR Validation

Candidate genes of interest harbouring the rare CNVs were validated by quantitative Real-Time PCR (qPCR) SyBr Green assay. Primers were designed using Primer3 (http://frodo.wi.mit.edu/primer3/) and checked with UCSC Genome Browser. Detailed information of the primer designed is shown in S1 Table. Normalization to the control gene Forkhead Box P2 (FOXP2) (primers: 5'-TGACATGCCAGCTTATCTGTTT-3' and 5'-GAGAAAAGCAATTTTCACAGTCC-3') was used to give an estimate of copy number.

## Results

### Clinical demographic data

**Table 1** shows the clinical and phenotypic data of the recruited subjects in the stage 1 study. Forty-four subjects were diagnosed as hypertension with LVH (denoted herein as case), while 72 were hypertension without LVH (denoted herein as control). There was no significant difference between the case and control groups with regards to ethnicity, age, systolic and diastolic blood pressure. As expected, significant differences were observed between case and control groups with regards to LV mass, IVSD and LVMI, indicating well characterized samples between cases and controls. BMI showed a significant difference between the case and controls, as expected. Males are significantly higher in case group than the females in our study cohort. This is in line with the previous studies, suggesting gender differences in the pathogenesis of LVH [26].

**Table 1:** Description of the study population.

	Case	Control	Total	P-value
N	44	72	116	
Ethnicity				
Malay	39	59	113	0.579
Chinese	3	7		
Indian	1	4		
Gender				
Male	37	48	115	0.028*
Female	6	24		
Age (years)	53.79	52.76		0.398
BMI (kg/m^2^)	28.12	26.22		0.040*
Systolic blood pressure (mmHg)	155.58	149.88		0.168
Diastolic blood pressure (mmHg)	93.16	94.53		0.846
LV mass (g)	239.17	162.07		<0.001*
IVSD	1.25	0.92		<0.001*
LVMI	143.89	92.71		<0.001

Abbreviations: BMI, body mass index; LV, left ventricular; IVSD, interventricular septum diastolic; LVMI, left ventricular mass index. Case, hypertension with LVH; control, hypertension without LVH.

* significantly different at P < 0.05

### Characterization of CNVs

**Fig 1:**
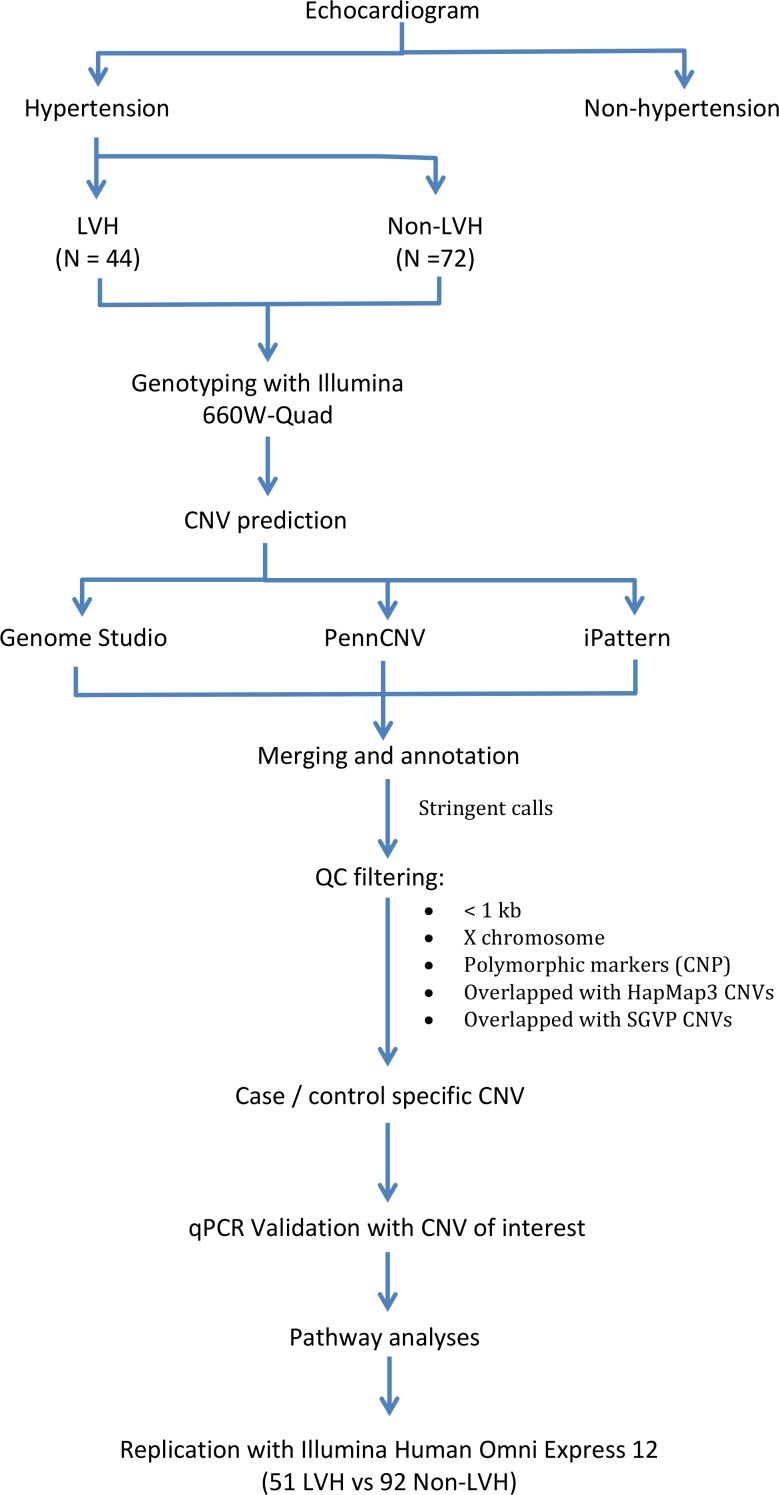
Experimental workflow describes the procedure for identifying CNV in subjects with hypertension related LVH.

**Fig 2:**
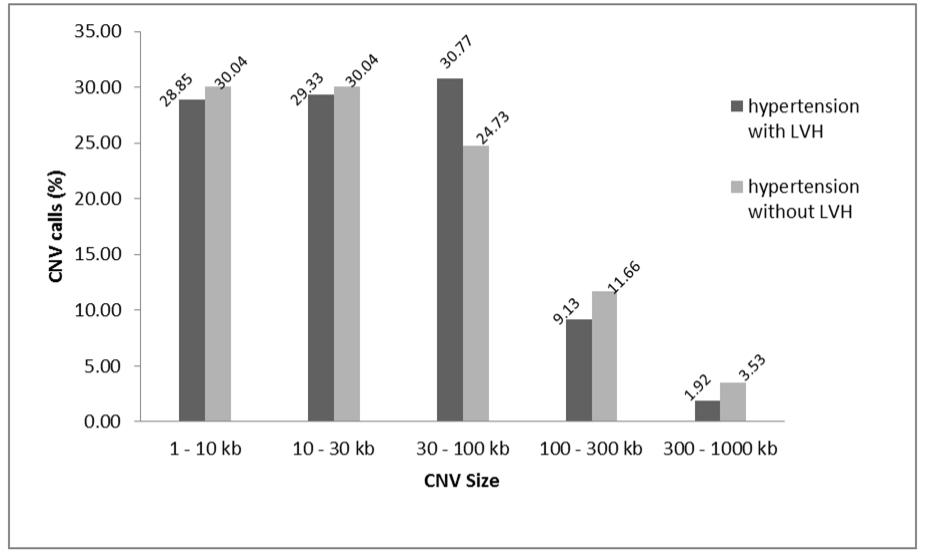
Histogram displaying the length distribution (in percentage) of the CNVs calls.

**Fig 1** describes the experimental workflow for identification of CNV specific to subjects hypertension related LVH. Of the 116 hypertensive subjects studied, 29,472, 33,473 and 33,203 CNVs were called by CNVPartition, PennCNV, and iPattern, respectively (Table 2). A total of 22,337 CNVs were successfully merged with at least two algorithms (referred as “stringent calls”), corresponding to an average of 202.9 CNVs per genome, with a median size of 3,893 bp (average size 19,428 bp) (**Table 2**). The number of CNVs per genome was relatively higher than previous reports [22,27,28] but in line with Pinto et al. (2011) which reported an average of 240 calls genome for the Illumina 660W platform. This could be due to several reasons: (i) different platforms utilized for CNV detection and its resolution; ii) levels of QC stringency applied during CNV call; iii) the algorithms applied when performing CNV call; [29]. Of particular note, the Illumina 660W is a platform seeded with probes to allow detection for common CNVs, thus expected to have higher sensitivity. A total of 1,973 CNVs unique to our study subjects remained after applying the QC filtering steps by excluding known polymorphic CNVs (copy number polymorphism, CNP), calls adjacent to telomere and centromere regions, and those reported in DGV and SGVP. The pre-defined criterion of 50% reciprocal overlapped was applied, whereby a CNV that was at least 50% unique by length when compared to every CNV in the control datasets was taken as putative novel [23]. This included 851 CNVs in cases (74 gain, 777 loss) and 1,122 in controls (162 gain, 960 loss). The length distribution of the CNVs observed is shown in **Fig 2**.

**Table 2:** General characteristics of CNV among the 116 genomes of hypertension subjects from Peninsular Malaysia.

	CNVpartition	PennCNV	iPattern	Merged*
**Total CNV count:**				
Gain	1,687	10,134	10,152	1,917
Loss	27,785	23,339	23,051	21,420
Total	29,472	33,473	33,203	23,337
**Average number per genome:**				
Gain	14.7	88.1	88.3	16.7
Loss	241.6	202.9	200.4	186.3
Total	256.3	291.1	288.7	202.9
**Size (bp):**				
Min	1,001	1,000	1,000	1,004
Max	3,908,024	935,550	1,015,980	1,015,981

Abbreviations: CNV, copy number variant; bp, base pair.

* Merged: stringent CNV calls by at least 2 out of 3 algorithms applied

qPCR was performed as an independent technical validation. *FOXP2* was used as the reference gene. Results of the qPCR validation are shown in **S1 Table**. Seven out of 9 (77%) of the loci validated were positive calls. Candidate genes underlying these CNVs were excluded in the subsequent pathway analysis.

### Case- and control-specific CNV

**Fig 3:**
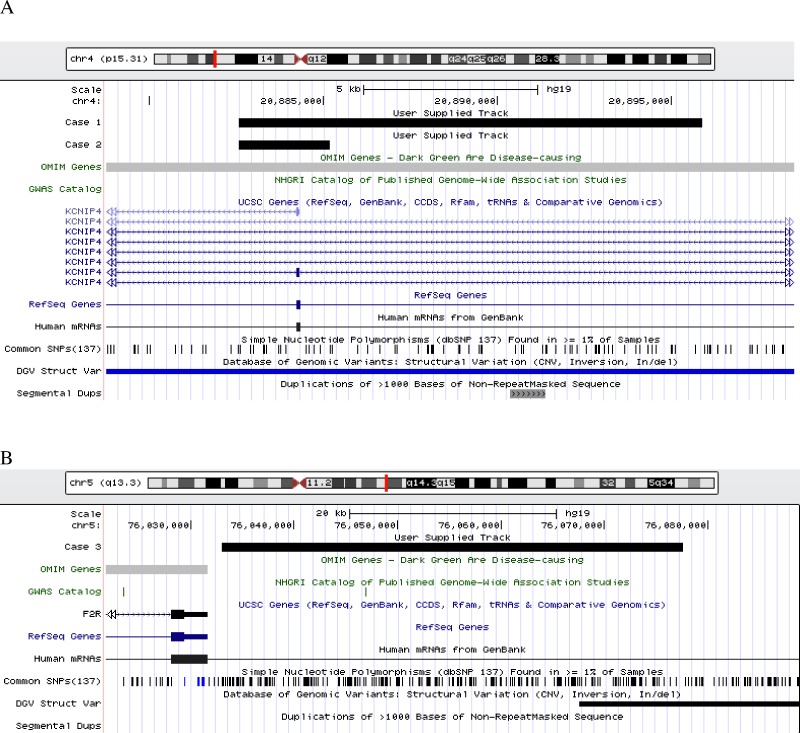
UCSC Genome browser view of rare CNV in the regions (A) KCNIP4; (B) F2R, as designated by black bar. Figures produced with custom tracks listing CNV calls and uploaded to http://genome.ucsc.edu. The hypertensive LVH cases are designated as black bars.

We further identified from the dataset, 208 CNVs specific to cases (35 gains; 173 losses) and 283 specific to controls (75 gains; 208 losses), which corresponded to an average of 4.72 and 3.93 CNV per genome in case and control groups, respectively. The overall CNV length distribution between case and control was similar between the cases and controls (average length 50,152.94 bp vs 53,194.88 bp) (P = 0.163) suggesting that CNV length may have minimal impact to LVH development. Recurrent CNVs for gene *LOC348021*, was observed in 3 cases; while *CDH15* and *KCNIP4* were observed in 2 cases, respectively (**Fig 3**, **S2 Table**). We performed targeted dosage analysis with qPCR for these candidate genes in additional 36 cases recruited from the same cohort, but no CNVs were detected.

### Gene Ontology and Pathway Analysis

Gene ontology and pathway analyses were then performed on the genes specific to case using 3 approaches.

### DAVID pathway analysis

We first performed analysis with DAVID. It is a publicly available threshold-based ontology analysis, which provides extensive option for interrogation of approximately 40 databases including KEGG, Interpro etc [30]. Results presented by DAVID are based upon the use of functional annotation clustering and the top five single terms among clusters will be reported.

Forty two case-specific genes were found involve in “ion binding” (GO:0043167) (P = 0.029); whereas “translational initiation” (GO:006413) (P = 0.003) and “translational factor activity / nucleic acid biding” (GO:0008135) (P = 0.037) were presented as the most significant GO processes (**S3 Table**).

### Ingenuity Pathway Analysis (IPA)

IPA is a JAVA based commercialized web-based system. Its annotations of genes and pathways are based on its own database built on the findings from various literatures and publicly available databases including GO and Entrez Gene. Enrichment analyses of IPA are carried out using the right-tailed Fisher’s exact test and Benjamini-Hochberg multiple testing corrections [30].

**Fig 4:**
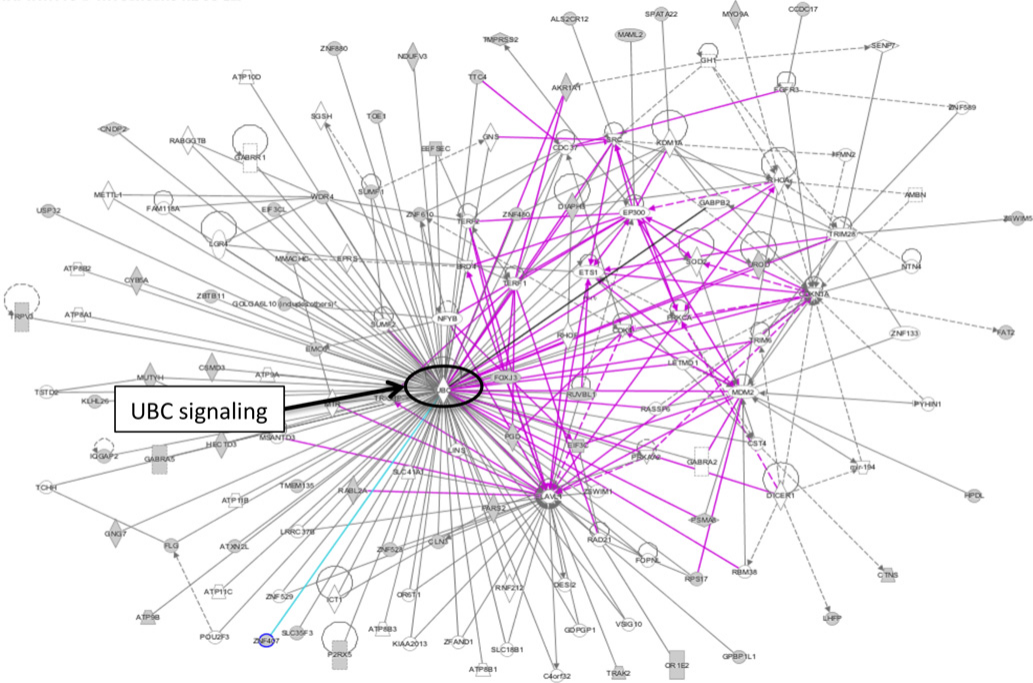
Network analyses of rare CNVs in the cases from stage-1 analysis. Network was generated by Ingenuity Pathway Analysis by merging up the top canonical pathways with default settings. Network analysis revealed UBC signalling as the major interacting network.

The “case-specific” genes identified in this study were enriched in diseases involved in inflammatory response (P = 3.43E-04), cancer (P = 2.87E-03), respiratory disease (P = 2.87E-03) connective tissue disorder (P = 5.56E-03) and skeletal and muscular disorders (P = 5.56E-03) (S4 Table). Functional enrichment analyses of IPA revealed top two most significant enriched genes in cell-to-cell signalling interaction (P = 3.43E-04–4.65E-02), and tissue development (P = 1.04E-04–4.91E-02) (**S3 Table**). Network analysis revealed UBC signalling as the major interacting network (**Fig 4**).

### GeneGO MetaCore analysis

GeneGO Metacore pathway analysis is a commercial web-delivered application based on unique, cur-rated database from Thompson Reuters containing approximately 20 validated functional ontologies that can be used for filtering and enrichment, as well as interactive canonical pathways capturing ~200,000 pathways.

Metacore analysis identified 69 genes most significantly involved in heart development (P = 1.47E-57); whereas another two most relevant tissue groups involved were skeletal muscle (67 genes; P = 1.64E-52), and smooth muscle (66 genes; P = 7.72E-52). The GO process were most significant with: “regulation of purine nucleotide metabolic process” (P = 1.28E-06), “regulation of neuron migration” (P = 5.00E-06), “intracellular signal transduction” (P = 1.22E-05), “negative regulation of renin secretion into blood stream” (P = 4.03E-05), and “hydrogen peroxide catabolic process” (P = 4.35E-05).

**Fig 5:**
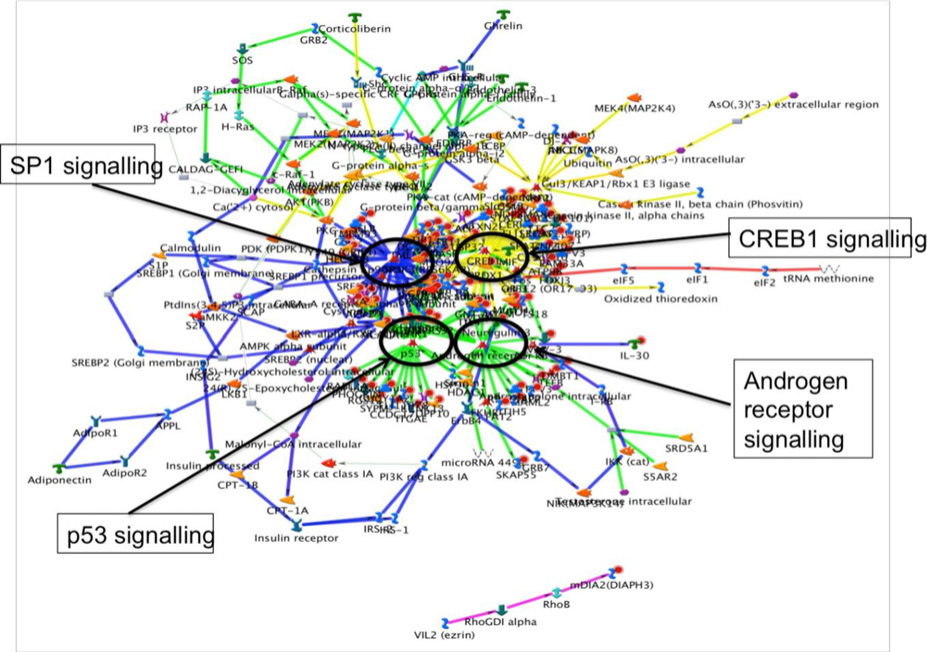
Network analyses of rare CNVs in cases from stage-1 analysis. Network was generated by GeneGo Metacore pathway analysis programme by merging the top canonical pathways with default settings.

We then predicted the most probable networks that may involve in LVH pathogenesis by pooling the 3 most enriched pathways from Metacore canonical network analysis. It was found that the gene-set was, directly or indirectly related to the transcription factors Sp1, p53 and CREB1, and androgen receptor signalling cascades (**Fig 5**).

### Replication

The subsequent replication with an independent group of samples was carried out using Illumina Omni Express SNP microarray, and the CNV was called using the stringent criteria pipeline is as described above, except that the CNVpartition was replaced by QuantiSNP, owing to its sensitivity in identifying CNVs from this platform (only a total of 829 CNVs were identified using CNVpartition from Genome Studio).

Rare CNVs were detected in 128 out of the 148 subjected included in the replication study. An average of 11 CNVs per genome were identified, ranging from 2 to 27 CNVs per genome (**Table 3**). The lower number of CNV detected in the replication stage is expected, since Omni Express was not designed for the detection of CNV, unlike the 660W which was a hybrid platform (i.e. seeded with probes for common CNV detection), thus lower power in CNV detection. A total of 303 CNVs unique to our study subjects remained after applying the QC filtering steps as mentioned in the methodology. These included 111 CNVs specific in cases (55 gain, 56 loss; 2.52 CNVs per genome) and 113 control specific CNVs (80 gain, 112 loss; 1.35 CNVs per genome) (P = 0.1895) (**S6 Table**). qPCR validation on 5 randomly selected CNVs identified in this replication stage revealed 100% positive calls (**S1 Table**).

**Table 3:** General characteristics of CNV among the 128 genomes of hypertension subjects from Peninsular Malaysia in the replication study.

	QuantiSNP	PennCNV	iPattern	Merged*
**Total CNV count:**				
Gain	1,223	906	3,906	644
Loss	1,253	4,304	6,015	794
Total	2,476	5,210	9,920	1,438
**Average number per genome:**				
Gain	9.6	7.1	30.5	5.0
Loss	9.8	33.6	47.0	6.2
Total	19.3	40.7	77.5	11.2
**Size (bp):**				
Min	1,031	1,030	1,058	1,038
Max	2,388,965	1,740,417	2,410,163	1,740,417

Abbreviations: CNV, copy number variant; bp, base pair.

* Merged: stringent CNV calls by at least 2 out of 3 algorithms applied

Notably, *SUMF1*, *F2R* and *IQGAP2* (found in the case group) and *ACSF3* (found in the control group) identified in the initial study were replicated in the replication cohort, suggesting potential role of these genes in pathophysiology of hypertension related LVH.

Gene Ontology and pathway enrichment analyses were carried out using DAVID and IPA. This analysis was carried out in 2 stages: (i) we first assessed the gene list revealed from the replication cohort; (ii) subsequently the gene list was combined from the “Illumina 660W” cohort and the “Illumina Omni Express” replication cohort (**S7 Table**). DAVID pathway analysis revealed the “case-specific” genes significantly enriched in EGF signalling (IPR:013111) (P = 0.0179); and “activation of protein kinase activity (GO:0032147)” (P = 0.0064). When the two stages were combined, EGF-like domain (IPR:006210) (P = 1.36E-03) remained the most significant enrichment; whilst the category “ion-binding” (GO:0043167) comprised the most number of genes (P = 0.040; 53 genes).

**Fig 6:**
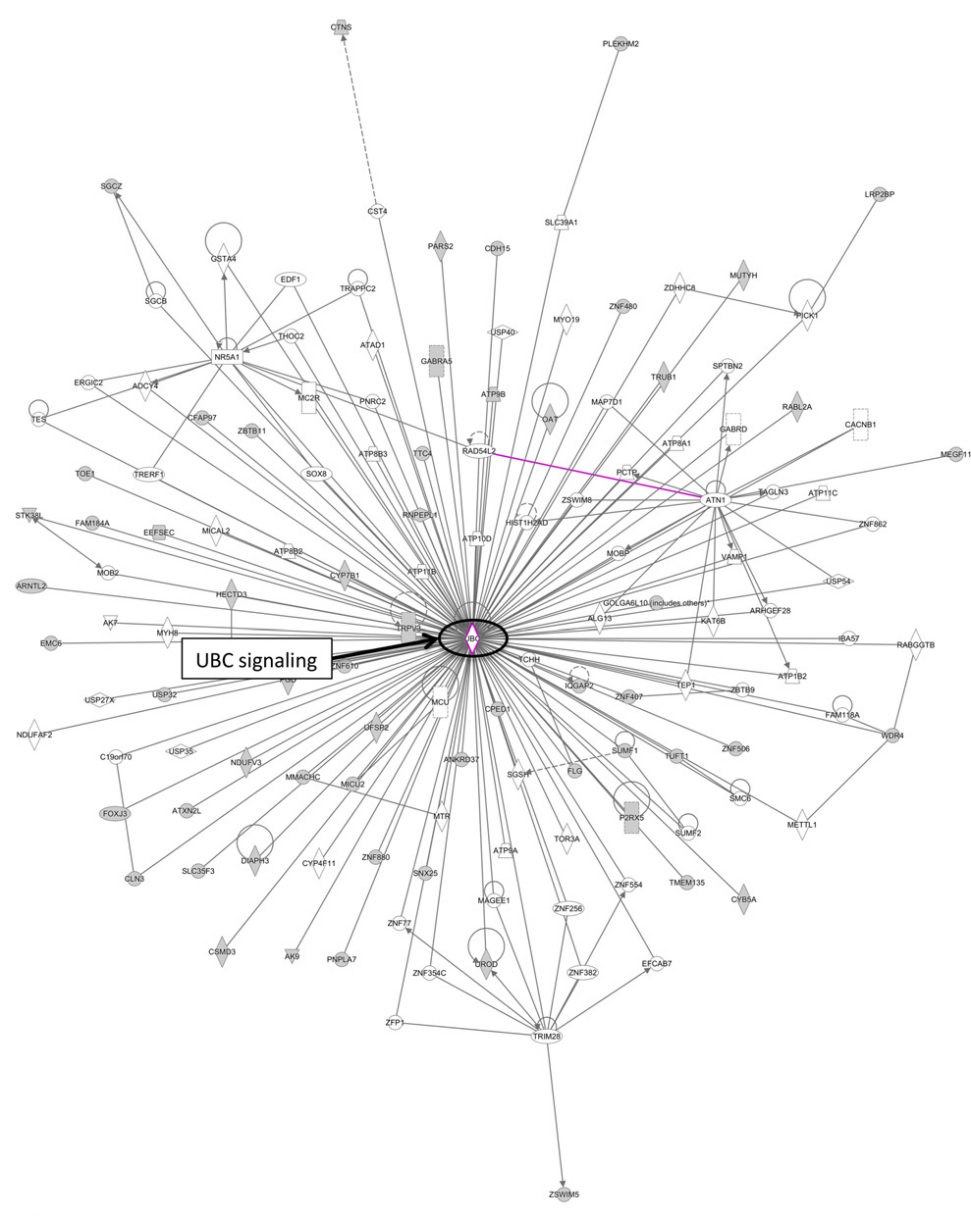
Network analyses of rare CNVs in cases. Network was generated by Ingenuity Pathway Analysis by merging up the top canonical pathways with default settings. Network analysis revealed UBC signalling as the major interacting network.

IPA analyses revealed significant enrichment in diseases related to infectious diseases (P = 7.73E-07–4.77E-02) and respiratory diseases (P = 7.73E-02–4.10E-02), suggesting disease mechanism may be related to immune inflammatory response, in line with the finding during the earlier stage (**S4 Table**). Interestingly, the most significant enrichment on physiological system development and function was “cardiovascular system development and function” (P = 7.48E-04–3.77E-02) (**S7 Table**). When merging the top 3 networks from IPA, Ubiquitin C (UBC) related signalling appeared to be the major network (**Fig 6**), and remained as the major interacting network analysis when gene lists from both stages were combined (**Fig 5**).

We note that some of the genes observed might not have any direct functional involvement with LVH or hypertension. However, collective findings from this study was connected though via biological interactions with numerous genes, many of which are known to be functionally involved in cardiac phenotypes in “foetal gene programmes” [31–33].

To ensure that the pathways and ontologies identified are unique in case, we performed a pathway analysis on the candidate genes identified in the control groups using DAVID. Apparently none of the pathways or ontologies identified were similar to the case groups (**S9 Table**).

## Discussion

In this study we applied an alternative approach for discovery of candidate genes involved in hypertension related-LVH. To our knowledge, this is the first report to evaluate the impact of rare CNVs on hypertension related-LVH conducted in the Southeast Asia population. We have provided further supporting evidence to show that rare CNV may have impact on common and complex disease susceptibility [22,27,34]. In addition our results also supported the previously reported signalling pathways for the development of LVH in the hypertensive subjects [5,31,35].

Several candidate genes were of interest and postulated to play a role in the LVH development. The coagulation factor II, *F2R*, is a G-protein coupled receptor family member (**Fig 3**, **S2 Table**). This gene was thought to function as a transmembrane receptor involved in the regulation of thrombotic response (http://www.ncbi.nlm.nih.gov/gene/2149), and may play a role in platelet activation and vascular development. Studies found that *F2R* interacts with *IL-6* in the susceptibility of myocardial infarction [36,37], and coronary heart disease in hypertensive patients [38]. However its role in the inflammatory mechanism of cardiac hypertrophy is not well understood. *IQGAP2*, a neighbouring gene of *F2R*, binds with CALM1 [39] and it regulates cell morphology and motility via interactions with components of cytoskeleton, cell adhesion and several other signalling molecules (http://www.ncbi.nlm.nih.gov/gene/10788), hence believed to be involved in the process of cardiac development. A recent study on whole genome exome sequencing reported a mutation of *IQGAP2* that caused LVH (Zhi et al., 2012). Both *F2R* and *IQGAP2* were found recurrent in our replication cohort. *KCNIP4*, a member of the voltage-gated potassium (Kv) channel-interacting protein family, has been reported to be associated with ischemic heart disease [40]. It was found to be associated with LVMI amongst the isolated Amish cohort in a genome-wide association study, though the signal was not significant in subsequent replication study [41]. *KCNIP4* is essential to the low repetitive firing and back propagation of action potential in neurons and shapes the action potential in the heart [42].

We observed several recurrent case specific CNVs, and attempted to further replicate these CNVs with additional 36 independent samples with qPCR on top of the 148 samples replicated with Illumina Omni Express. None of these samples however, were copy number variable, thus suggesting that these case-specific CNVs may be at a very low frequency. This postulation is indeed reasonable, considering that most rare / *de novo* CNVs occur less than 1% in a population [43–45].

Research of life sciences has shifted from gene identification to gene annotations including functions, interactions and the involvement of pathways [22,30,46–48]. Our exploratory data support the findings implicating the immune system and inflammation pathways in the aetiology of stressed induced cardiac hypertrophy [31,49]. At present, there is no single specific signalling pathway that could explain entirely the functional changes leading to cardiac hypertrophy. However, a number of transcription factor signalling pathways have been suggested [50]. Among these, the involvement of cAMP response element binding protein 1 (CREB1) transcription factor signalling as an important regulator in cardiac hypertrophy has been well acknowledged [14,51,52].

Specificity protein 1 (SP1) transcription factor and its signalling pathway is a major component during the foetal stage of cardiac development in human. It was identified as a transcriptional regulatory mechanism in the reduction of foetal metabolic programme during pressure overload induced cardiac hypertrophy [32].

Transcription factor p53 and its signalling are known to mediate apoptosis induced by multiple stresses [53], and crucially involved in cardiac hypertrophy [54].

The influence of androgen receptor signalling in cardiac hypertrophy has been proposed earlier [55–57], either by acting directly on the heart or by affecting the vascular system [58–61]. However, little is known about its role and the underlying molecular mechanism in cardiac hypertrophy as findings remain contradicting [28,55,57,62]

The Ubiquitin C (UBC) via its ubiquitination activities has been reported to play numerous physiological functions including that causing hypertrophic response [63–65]. The promotion of ubiquitination activities via expression of Atrogin-1 (*FBXO32*) and repression of calcineurin A apparently leads to inhibition of cardiac hypertrophy [66].

Numerous studies have provided evidence that induction of Ang II promotes the growth of cardiomyocytes via transactivation of EGF signalling subsequently activation of MAPK signalling [67]. It is interesting to note that transactivation EGF receptor signalling by AT1R activates CREB1 [68,69].

Several potential limitations have been identified in this study. First, relatively a small sample size, therefore statistical and functional analyses could not be carried out. Secondely, rare CNVs (defined as frequency <1%) could hardly reach a significant number eligible for statistical analyses [70]. Therefore it is difficult to replicate the findings in independent studies. Thirdly, false negative results probably due to stringent filtering criteria for CNV calls, thus potentially limiting discovery of novel and informative findings. On top of that, inclusion of smaller CNVs in this study. We included all CNVs sized >1 kb instead of >30 kb as practiced by most reports [20,22,27,28,34,71,72]. We believed that selection of larger CNVs might introduced potential bias to the impact of CNVs in disease pathogenesis especially in complex diseases with lower inheritability such as hypertension related LVH, and presuming that smaller CNV may well contribute equal impact with the larger ones especially when exons or splicing sites of a gene is being disrupted by CNV breakpoints. However, smaller CNVs causes higher false discovery rate due to poor signal to noise ratio, thus data should handle with caution. Lastly, The biological replication of the rare CNV was carried out using a different platfrom (Illumina OmniExpress, which was not meant for CNV detection), leading to a significant drop in CNVs calls per sample. (and decrease in unique CNVs), indicating that the replication was not as powerful as it was expected, therefore the lowered the power of CNV detection. However, this does not increase the false positive rate of this study, therefore the results reported are considerable accurate.

Despite this, our data was analysed with a most stringent QC criteria by comparing with several datasets including DGV, HapMap3 and the SGVP, involving more than 1,300 samples, and were subsequently selectively validated. As such, our CNV call is considered reliable. However, we acknowledge the potential constraint of these datasets as they were genotyped with different platforms therefore further interpretation of the finding should be taken cautiously.

It should be noted that this study however does not attempt to prove the involvement with the disease of any specific variants or even any specific genes or pathways. Rather, it provides insights that common diseases like hypertension related LVH can be affected by rare CNVs that influence or disrupt the underlying genes in the relevant pathways. In other word, rare CNVs identified in this study are not proven to be the causative variants, rather they may contribute as a portion of risk factor to the common disease susceptibility such as hypertension-related LVH. An independent cohort with larger number of samples is crucial to warrant the findings of this study. Functional studies characterizing the role in cardiac development of genes within these rare CNVs are a priority to illuminate the mechanisms of LVH development in hypertensive patients.

In summary, this study delivers two major messages. First, we show that rare variant plays a role in the susceptibility of common and complex diseases such as LVH. Indeed, using the rare CNV strategy, we have demonstrated further supporting evidence of the previously identified signalling pathways leading to cardiac hypertrophy. In particular, collective results of this study further support the activation of foetal cardiac gene programme during cardiac hypertrophy. Second, LVH is a complex event that depends on the activation of different signalling pathways. The finding of this work may eventually lead on to work concerning differences in response to drugs, which can prevent the development of LVH in patients with hypertension.

## Supporting Information

S1 Table Candidate genes primers sequences for SyBr Green qRT-PCR assay. (DOC)

S2 Table Case- and control specific CNVs identified in the 116 hypertension related LVH subjects studied. Chr, chromosome; hg18, human genome assembly 18 (March 2006). Dashes indicate that no gene is involved or disrupted by CNV breakpoints. Highlighted are genes with evidence for cardiovascular involvement.
(DOC)

S3 Table Gene ontology and pathway analyses identified in hypertension-related LVH. (DOC)

S4 Table Top significant disease groups identified by Ingenuity (IPA) enrichment analysis. (DOC)

S5 Table Top significant tissue groups identified by GO MetaCore enrichment analysis. (DOC)

S6 Table Case- and control specific CNVs identified in the 116 hypertension related LVH subjects in the replication study. Chr, chromosome; hg18, human genome assembly 18 (March 2006). Dashes indicate that no gene is involved or disrupted by CNV breakpoints. Highlighted are genes that are identified in the earlier stage of the study.
(DOC)

S7 Table Gene ontology and pathway analyses identified in the hypertension-related LVH from the replication study. (DOC)

S8 Table Top 5 networks identified by Ingenuity (IPA) from the replication study. (DOC)

S9 Table Gene ontology and pathway analyses identified using DAVID in the hypertension patients without LVH (denoted as controls) from (i) Illumina 660W; (ii) Illumina Omni Express; (iii) combination from Illumina 660W and Illumina Omni Express datasets.
(DOC)
