# Supplementary material for: Rare Copy Number Variants Identified Suggest the Regulating Pathways in Hypertension-Related Left Ventricular Hypertrophy
Source: PLoS One. 2016 Mar 1;11(3):e0148755. doi: 10.1371/journal.pone.0148755 (PMC4773219; doi:10.1371/journal.pone.0148755)
Supplement: S1 Table — (DOC) [file pone.0148755.s001.doc]

**S1 Table. Candidate genes primers sequences for SyBr Green qRT-PCR assay**

| Locus name | Primer sequence | Expected  amplicon  size (bp) | | | Annealing temp (^o^C) | Microarray CNV calls (gain/loss) | Unrounded Copy Number | Rounded Copy Number |
| --- | --- | --- | --- | --- | --- | --- | --- | --- |
| IL27RA | Forward: 5’AGATGTGTGGGTATCAGGGAAC3’  Reverse: 5’CGTGCAGAGAAATGTAACTGGT3’ | | 102 | 60 | | Gain | 2.749 | 3 |
| F2R | Forward: 5’AACCCTGCTCGAAGGCTACTAT 3’  Reverse: 5’ GACACATAACAGACCGTGGAAA 3’ | | 96 | 60 | | Gain | 4.298 | 4 |
| NRG3 | Forward: 5' ATGCAGAGACCTTGTGTGCTTA 3’  Reverse: 5' ACAAACTCTTCCTGAACCTCGT 3’ | | 91 | 60 | | Loss | 2.012 | 2 |
| ITPR3 | Forward: 5' TGGCCTTTACCTATGCTCTCA 3’ Reverse: 5' AGGTGGTAAGCAAATTGGTGTT 3’ | | 92 | 60 | | Loss | 1.242 | 1 |
| EIF2B3 | Forward: 5'-CATGGCTAATGAAGCAGACTTG 3’ Reverse: 5'-ACAAGGAGAATTGCCAGAAGAA 3’ | | 99 | 60 | | Gain | 2.671 | 3 |
| KCNIP4 | Forward: 5' CTCACGTTCACCTTAGTTGCAGA 3’ Reverse: 5' CATGCGTTAAAGATTACAGCAGGT 3’ | | 102 | 60 | | Loss | 0.731 | 1 |
| APOB48R | Forward: 5’ ACACCCTGGAAGAGCTTTGAT 3’  Reverse: 5’ ACAGGCACATGAAGAGAGTCAAT 3’ | | 92 | 60 | | Loss | 1.196 | 1 |
| FAT2 | Forward: 5' AGGATGTCAACGACAACTCTCC 3’ Reverse: 5' GATCAGAGGCATCCAGAAATGT 3’ | | 108 | 60 | | Loss | 2.086 | 2 |
| WDR4 | Forward: 5’ GGGACGATTCCAGTAGAAATGA3’  Reverse: 5’ CAAGAACCAAAGTCACAGTTCG 3’ | | 103 | 60 | | Gain | 3.615 | 4 |
| PTPRM | Forward: 5’ CTTCATTACAGCAGGCATGTTC3’  Reverse: 5’ ATGAGGTTGTTGTCAAAGTAGCC3’ | | 102 | 60 | | Gain | 3.459 | 3 |
| FHIT | Forward: 5’ GGTACTCCTTTGCTTGCAGAAA 3’  Reverse: 5’ CCACTCCAGACATCTCACCATT 3’ | | 116 | 60 | | Loss | 1.017 | 1 |
| SLCO1B1  SORBS2  STK38L | Forward: 5’ TCCAAGGCAAGACATATGCTCA 3’  Reverse: 5’ TGACCTTTCACTTGCCTTGTTC 3’  Forward: 5’ AAACTGGAAATTTAGGTGCGGG 3’  Reverse: 5’ GAGCCCTCTGGTAGCAGTATCT3’  Forward: 5’ ACAGAACCGGACTACAAATCCA 3’  Reverse: 5’ ACTTCCCAGCTTTCATGTAGGT 3’ | | 97  92  112 | 60  60  60 | | Loss  Loss  Gain | 1.360  0.945  3.871 | 1  1  4 |
