# Supplementary material for: Rare Copy Number Variants Identified Suggest the Regulating Pathways in Hypertension-Related Left Ventricular Hypertrophy
Source: PLoS One. 2016 Mar 1;11(3):e0148755. doi: 10.1371/journal.pone.0148755 (PMC4773219; doi:10.1371/journal.pone.0148755)
Supplement: S2 Table — Chr, chromosome; hg18, human genome assembly 18 (March 2006). Dashes indicate that no gene is involved or disrupted by CNV breakpoints. Highlighted are genes with evidence for cardiovascular involvement. (DOC) [file pone.0148755.s002.doc]

**S2 Table. Case- and control specific CNVs identified in the 116 hypertension related LVH subjects studied. Chr, chromosome; hg18, human genome assembly 18 (March 2006). Dashes indicate that no gene is involved or disrupted by CNV breakpoints. Highlighted are genes with evidence for cardiovascular involvement.**

| **Chr Cytoband** | **Case/ control** | **Start** | **End** | **Size** | **CNV freq *** | **CNV type**  **(gain/loss)** | **Genes involved** | **Disrupted genes** |
| --- | --- | --- | --- | --- | --- | --- | --- | --- |
| 1p13.3 | Case | 108,199,917 | 108,206,873 | 6,957 |  | Loss | *VAV3* | *-* |
| 1p22.2 | Case | 88,867,128 | 88,870,440 | 3,313 |  | Loss | *-* | *-* |
| 1p31.1 | Case | 76,878,790 | 76,885,961 | 7,172 |  | Loss | *-* | *-* |
| 1p32.3 | Case | 54,896,976 | 55,021,290 | 124,315 |  | Gain | *C1orf175, TTC4, TTC22, PARS2* | *C1orf175, TTC22* |
| 1p34.1 | Case | 45,091,339 | 45,925,790 | 834,452 |  | Gain | *HECTD3, hCG_1820661, GPBP1L1, TOE1, EIF2B3, PRDX1, MUTYH, ZSWIM5, MMACHC, AKR1A1, CCDC163P, RPS15AP10, UROD, NASP, CCDC17, HPDL, TESK2* | *EIF2B3* |
| 1p34.2 | Case | 42,452,914 | 42,474,931 | 22,018 |  | Loss | *FOXJ3* | *FOXJ3* |
| 1p34.3 | Case | 38,856,998 | 38,865,078 | 8,081 |  | Loss | *-* | *-* |
| 1p36.13 | Case | 17,549,319 | 17,557,761 | 8,443 |  | Loss | *PADI4* | *PADI4* |
| 1p36.13 | Case | 19,246,192 | 19,259,929 | 13,738 |  | Loss | *-* | *-* |
| 1p36.21 | Case | 15,874,175 | 15,883,925 | 9,751 |  | Loss | *PLEKHM2* | *PLEKHM2* |
| 1p36.22 | Case | 10,389,661 | 10,406,043 | 16,383 |  | Loss | *PGD* | *PGD* |
| 1q21.3 | Case | 150,501,668 | 150,552,991 | 51,324 |  | Gain | *FLG* | *FLG* |
| 1q31.1 | Case | 186,741,046 | 186,788,268 | 47,223 |  | Loss | *-* | *-* |
| 1q31.1 | Case | 186,746,469 | 186,887,565 | 141,097 |  | Loss | *-* | *-* |
| 1q31.1 | Case | 188,415,821 | 188,453,010 | 37,190 |  | Loss | *FAM5C* | *-* |
| 1q31.2 | Case | 189,113,886 | 189,196,044 | 82,159 |  | Loss | *-* | *-* |
| 1q32.1 | Case | 204,495,676 | 204,661,633 | 165,958 | 0.017 | Loss | *CTSE, SRGAP2* | *CTSE, SRGAP2* |
| 1q41 | Case | 216,052,051 | 216,314,883 | 262,833 |  | Loss | *SPATA17* | *SPATA17* |
| 1q41 | Case | 220,377,302 | 220,444,691 | 67,390 |  | Loss | *-* | *-* |
| 1q41 | Case | 220,393,433 | 220,447,047 | 53,615 |  | Loss | *-* | *-* |
| 1q42.2 | Case | 232,382,561 | 232,386,873 | 4,313 |  | Gain | *SLC35F3* | *-* |
| 1q43 | Case | 236,816,026 | 236,818,560 | 2,535 |  | Loss | *-* | *-* |
| 1q44 | Case | 244,334,189 | 244,624,189 | 290,001 |  | Loss | *~~SMYD3~~* | *~~SMYD3~~* |
| 2p11.2 | Case | 89,079,714 | 89,113,055 | 33,342 |  | Gain | *-* | *-* |
| 2p24.2 | Case | 17,449,692 | 17,457,764 | 8,073 |  | Loss | *-* | *-* |
| 2p25.3 | Case | 1,672,042 | 1,689,441 | 17,400 |  | Gain | *PXDN* | *PXDN* |
| 2q14.1 | Case | 114,055,115 | 114,127,652 | 72,538 |  | Gain | *RABL2A, WASH2P, MGC13005, RPL23AP7* | *-* |
| 2q14.1 | Case | 115,236,675 | 115,251,611 | 14,937 |  | Loss | *~~DPP10~~* | *-* |
| 2q21.2, 2q21.3 | Case | 134,731,817 | 134,843,944 | 112,128 |  | Loss | *MGAT5* | *MGAT5* |
| 2q22.2 | Case | 142,938,475 | 143,235,708 | 297,234 |  | Gain | *-* | *-* |
| 2q32.1 | Case | 188,828,082 | 188,830,474 | 2,393 |  | Loss | *-* | *-* |
| 2q33.1 | Case | 201,925,161 | 201,988,385 | 63,225 |  | Loss | *TRAK2, ALS2CR12* | *TRAK2, ALS2CR12* |
| 2q36.1 | Case | 221,926,333 | 221,947,129 | 20,797 |  | Loss | *-* | *-* |
| 2q36.2 | Case | 224,999,036 | 225,002,142 | 3,107 |  | Loss | *-* | *-* |
| 2q36.2 | Case | 225,011,266 | 225,014,127 | 2,862 |  | Loss | *-* | *-* |
| 3p14.1 | Case | 69,249,046 | 69,260,603 | 11,558 |  | Loss | *LMOD3* | *LMOD3* |
| 3p26.1, 3p26.2 | Case | 5,466,322 | 5,538,371 | 72,050 |  | Loss | *-* | *-* |
| 3p26.2 | Case | 4,452,911 | 4,505,278 | 52,368 |  | Loss | *SUMF1* | *SUMF1* |
| 3q12.1 | Case | 100,302,740 | 100,378,340 | 75,601 |  | Loss | *-* | *-* |
| 3q12.3 | Case | 102,840,256 | 102,871,883 | 31,628 |  | Loss | *ZBTB11* | *ZBTB11* |
| 3q21.3 | Case | 129,300,645 | 129,361,561 | 60,917 |  | Gain | *EEFSEC, RUVBL1* | *EEFSEC, RUVBL1* |
| 3q23 | Case | 142,028,103 | 142,031,775 | 3,673 |  | Loss | *-* | *-* |
| 3q23 | Case | 142,028,103 | 142,031,775 | 3,673 |  | Loss | *-* | *-* |
| 3q24 | Case | 149,308,057 | 149,350,318 | 42,262 |  | Loss | *-* | *-* |
| 3q26.31 | Case | 175,680,795 | 175,685,302 | 4,508 |  | Gain | *-* | *-* |
| 3q26.33 | Case | 183,074,866 | 183,125,222 | 50,357 |  | Loss | *-* | *-* |
| 3q27.1 | Case | 185,172,683 | 185,197,161 | 24,479 |  | Loss | *ABCC5* | *ABCC5* |
| 3q27.3 | Case | 187,596,961 | 187,678,017 | 81,057 |  | Gain | *-* | *-* |
| 4p13 | Case | 42,441,381 | 42,456,190 | 14,810 |  | Loss | *-* | *-* |
| 4p15.31 | Case | 20,882,574 | 20,885,178 | 2,605 |  | Loss | *KCNIP4* | *-* |
| 4p15.31 | Case | 20,882,574 | 20,895,875 | 13,302 |  | Loss | *KCNIP4* | *-* |
| 4p16.1 | Case | 6,710,585 | 6,731,478 | 20,894 |  | Loss | *LOC93622* | *-* |
| 4p16.2 | Case | 3,308,836 | 3,313,273 | 4,438 |  | Loss | *RGS12* | *-* |
| 4q13.1 | Case | 64,779,730 | 64,795,145 | 15,416 |  | Loss | *-* | *-* |
| 4q21.1 | Case | 76,518,619 | 76,542,546 | 23,928 |  | Loss | *-* | *-* |
| 4q31.1 | Case | 140,794,951 | 140,828,775 | 33,825 |  | Loss | *MGST2* | *MGST2* |
| 4q31.23 | Case | 148,735,119 | 148,741,744 | 6,626 |  | Loss | *-* | *-* |
| 4q31.3 | Case | 154,972,328 | 154,982,349 | 10,022 |  | Loss | *-* | *-* |
| 4q33 | Case | 172,030,151 | 172,079,301 | 49,151 |  | Gain | *-* | *-* |
| 4q34.3 | Case | 178,205,572 | 178,415,154 | 209,583 |  | Loss | *-* | *-* |
| 5p14.1 | Case | 28,528,218 | 28,534,740 | 6,523 |  | Loss | *-* | *-* |
| 5p14.2 | Case | 23,938,905 | 23,945,676 | 6,772 |  | Loss | *-* | *-* |
| 5p14.2 | Case | 24,070,307 | 24,165,174 | 94,868 |  | Loss | *-* | *-* |
| 5p14.3 | Case | 20,813,443 | 20,870,460 | 57,018 |  | Loss | *-* | *-* |
| 5p15.1 | Case | 15,382,810 | 15,473,775 | 90,966 |  | Loss | *-* | *-* |
| 5p15.2 | Case | 12,554,892 | 12,794,662 | 239,771 |  | Loss | *-* | *-* |
| 5p15.33 | Case | 1,977,628 | 1,985,463 | 7,836 |  | Loss | *-* | *-* |
| 5q12.1 | Case | 60,630,100 | 60,721,514 | 91,415 | 0.017 | Gain | *ZSWIM6* | *ZSWIM6* |
| 5q12.1 | Case | 61,610,593 | 61,613,798 | 3,206 |  | Loss | *-* | *-* |
| 5q13.3 | Case | 74,435,622 | 74,471,080 | 35,459 |  | Loss | *ANKRD31* | *ANKRD31* |
| 5q13.3 | Case | 76,032,969 | 76,077,666 | 44,698 |  | Gain | *NCRUPAR, F2R, IQGAP2* | *IQGAP2* |
| 5q14.3 | Case | 87,414,783 | 87,423,328 | 8,546 |  | Loss | *-* | *-* |
| 5q14.3 | Case | 90,515,185 | 90,559,425 | 44,241 |  | Loss | *-* | *-* |
| 5q23.2 | Case | 123,881,433 | 123,937,399 | 55,967 |  | Gain | *-* | *-* |
| 5q33.1 | Case | 147,533,379 | 147,556,650 | 23,272 |  | Loss | *SPINK5L2* | *SPINK5L2* |
| 5q33.1 | Case | 147,533,379 | 147,600,363 | 66,985 |  | Loss | *SPINK6, SPINK5L2* | *SPINK5L2* |
| 5q33.1 | Case | 150,206,915 | 150,279,814 | 72,900 |  | Loss | *IRGM, ZNF300* | *IRGM* |
| 5q33.1 | Case | 150,923,162 | 150,936,978 | 13,817 | 0.026 | Loss | *FAT2* | *FAT2* |
| 5q34 | Case | 164,014,586 | 164,095,625 | 81,040 |  | Loss | *-* | *-* |
| 6p12.3 | Case | 46,412,204 | 46,429,048 | 16,845 |  | Loss | *-* | *-* |
| 6p21.1 | Case | 41,792,623 | 41,811,180 | 18,558 |  | Loss | *TFEB* | *TFEB* |
| 6p21.1 | Case | 41,802,750 | 41,811,180 | 8,431 |  | Loss | *TFEB* | *TFEB* |
| 6p21.32 | Case | 32,720,633 | 32,752,837 | 32,205 |  | Loss | *HLA-DQB1* | *-* |
| 6p22.2 | Case | 24,431,837 | 24,435,480 | 3,644 | 0.026 | Loss | *DCDC2* | *-* |
| 6q12 | Case | 69,255,558 | 69,312,072 | 56,515 |  | Loss | *-* | *-* |
| 6q22.31 | Case | 119,305,179 | 119,388,611 | 83,433 |  | Loss | *FAM184A* | *FAM184A* |
| 6q27 | Case | 167,081,561 | 167,084,561 | 3,001 |  | Loss | *RPS6KA2* | *-* |
| 7p21.1 | Case | 15,709,618 | 15,727,202 | 17,585 | 0.026 | Gain | *-* | *-* |
| 7p21.3 | Case | 11,887,071 | 11,907,573 | 20,503 |  | Loss | *-* | *-* |
| 7p21.3 | Case | 13,244,825 | 13,249,052 | 4,228 |  | Loss | *-* | *-* |
| 7q11.22 | Case | 66,388,434 | 66,411,301 | 22,868 |  | Gain | *PMS2L4, STAG3L4* | *STAG3L4* |
| 7q31.2 | Case | 115,688,657 | 115,743,365 | 54,709 |  | Loss | *-* | *-* |
| 7q31.31 | Case | 120,860,140 | 120,877,767 | 17,628 |  | Loss | *-* | *-* |
| 7q31.33 | Case | 125,145,741 | 125,198,081 | 52,341 |  | Loss | *-* | *-* |
| 7q36.3 | Case | 157,739,419 | 157,753,078 | 13,660 |  | Loss | *PTPRN2* | *-* |
| 8p23.3 | Case | 1,396,677 | 1,428,246 | 31,570 |  | Loss | *-* | *-* |
| 8q12.3 | Case | 63,450,041 | 63,484,232 | 34,192 |  | Loss | *NKAIN3* | *-* |
| 8q23.3 | Case | 114,125,769 | 114,160,908 | 35,140 |  | Loss | *CSMD3* | *-* |
| 8q23.3 | Case | 115,464,627 | 115,579,946 | 115,320 |  | Loss | *-* | *-* |
| 8q24.23 | Case | 138,194,379 | 138,198,738 | 4,360 | 0.017 | Loss | *-* | *-* |
| 8q24.23 | Case | 138,891,879 | 138,894,883 | 3,005 |  | Loss | *-* | *-* |
| 8q24.3 | Case | 144,319,775 | 144,365,087 | 45,313 | 0.026 | Loss | *-* | *-* |
| 9q21.13 | Case | 77,279,443 | 77,282,614 | 3,172 |  | Loss | *-* | *-* |
| 9q33.3 | Case | 128,560,455 | 128,563,728 | 3,274 |  | Loss | *-* | *-* |
| 9q34.11 | Case | 132,061,708 | 132,074,219 | 12,512 |  | Loss | *-* | *-* |
| 10p14 | Case | 7,748,939 | 7,772,329 | 23,391 |  | Loss | *ITIH5* | *ITIH5* |
| 10q22.1 | Case | 72,526,759 | 72,532,869 | 6,111 |  | Loss | *-* | *-* |
| 10q22.1 | Case | 72,526,759 | 72,563,345 | 36,587 |  | Loss | *-* | *-* |
| 10q23.1 | Case | 84,253,897 | 84,394,962 | 141,066 |  | Loss | *NRG3* | *-* |
| 10q23.32 | Case | 93,623,410 | 93,633,175 | 9,766 |  | Loss | *-* | *-* |
| 10q23.33 | Case | 96,844,531 | 96,866,686 | 22,156 |  | Loss | *-* | *-* |
| 10q26.13 | Case | 124,350,152 | 124,366,633 | 16,482 |  | Loss | *DMBT1* | *DMBT1* |
| 10q26.3 | Case | 133,073,717 | 133,090,094 | 16,378 |  | Loss | *-* | *-* |
| 11p14.3 | Case | 24,231,908 | 24,242,693 | 10,786 | 0.026 | Loss | *-* | *-* |
| 11p15.5 | Case | 1,389,894 | 1,410,849 | 20,956 |  | Gain | *BRSK2* | *-* |
| 11q14.2 | Case | 86,610,251 | 86,624,672 | 14,422 |  | Gain | *TMEM135* | *-* |
| 11q21 | Case | 95,639,866 | 95,642,934 | 3,069 |  | Loss | *MAML2* | *-* |
| 11q22.3 | Case | 106,291,638 | 106,310,908 | 19,271 |  | Loss | *GUCY1A2* | *-* |
| 13q12.11 | Case | 18,462,715 | 18,482,477 | 19,763 |  | Loss | *LOC348021* | *LOC348021* |
| 13q12.11 | Case | 18,462,715 | 18,492,024 | 29,310 |  | Loss | *LOC348021* | *-* |
| 13q12.11 | Case | 18,467,428 | 18,496,030 | 28,603 |  | Loss | *LOC348021* | *-* |
| 13q13.3 | Case | 38,877,197 | 38,880,960 | 3,764 |  | Loss | *LHFP* | *-* |
| 13q21.2 | Case | 59,099,927 | 59,324,889 | 224,963 |  | Gain | *DIAPH3* | *DIAPH3* |
| 13q21.31 | Case | 61,674,643 | 61,684,686 | 10,044 |  | Loss | *-* | *-* |
| 13q34 | Case | 111,898,056 | 111,903,200 | 5,145 | 0.017 | Loss | *-* | *-* |
| 13q34 | Case | 111,898,056 | 111,918,207 | 20,152 |  | Loss | *-* | *-* |
| 13q34 | Case | 113,081,290 | 113,100,471 | 19,182 | 0.026 | Loss | *-* | *-* |
| 14q11.2 | Case | 21,457,679 | 21,490,102 | 32,424 |  | Loss | *-* | *-* |
| 14q11.2 | Case | 21,953,018 | 21,986,244 | 33,227 |  | Loss | *-* | *-* |
| 14q31.1 | Case | 80,948,670 | 80,952,396 | 3,727 |  | Loss | *-* | *-* |
| 14q32.13 | Case | 93,514,235 | 93,534,242 | 20,008 |  | Loss | *C14orf48* | *C14orf48* |
| 14q32.33 | Case | 104,069,741 | 104,108,470 | 38,730 |  | Loss | *-* | *-* |
| 14q32.33 | Case | 105,149,724 | 105,181,959 | 32,236 |  | Loss | *-* | *-* |
| 14q32.33 | Case | 105,178,726 | 105,181,959 | 3,234 |  | Loss | *-* | *-* |
| 14q32.33 | Case | 105,460,562 | 105,786,123 | 325,562 |  | Gain | *ADAM6, KIAA0125* | *KIAA0125* |
| 15q12 | Case | 24,643,347 | 24,664,404 | 21,058 |  | Gain | *GABRA5* | *GABRA5* |
| 15q22.2 | Case | 57,454,284 | 57,502,958 | 48,675 |  | Gain | *-* | *-* |
| 15q22.2 | Case | 60,491,181 | 60,494,997 | 3,817 | 0.017 | Loss | *-* | *-* |
| 15q23 | Case | 70,112,649 | 70,174,733 | 62,085 | 0.017 | Loss | *MYO9A* | *MYO9A* |
| 15q25.2 | Case | 80,510,416 | 80,831,541 | 321,126 |  | Gain | *RPS17, GOLGA6L9, UBE2Q2P3, UBE2Q2P2, LOC80154, GOLGA6L10* | *GOLGA6L9, UBE2Q2P3, UBE2Q2P2* |
| 15q26.2 | Case | 92,685,528 | 92,689,407 | 3,880 |  | Gain | *MCTP2* | *MCTP2* |
| 15q26.3 | Case | 96,401,042 | 96,417,183 | 16,142 | 0.017 | Loss | *-* | *-* |
| 16p11.2 | Case | 28,302,045 | 28,490,716 | 188,672 |  | Loss | *EIF3CL, CCDC101, NUPR1, EIF3C, CLN3, IL27, APOB48R* | *EIF3CL, CCDC101, EIF3C* |
| 16p11.2 | Case | 28,325,247 | 28,450,882 | 125,636 |  | Loss | *APOB48R, CLN3, IL27* | *-* |
| 16p11.2 | Case | 28,658,918 | 28,742,869 | 83,952 |  | Gain | *ATXN2L* | *ATXN2L* |
| 16q13 | Case | 55,918,847 | 55,932,768 | 13,922 | 0.017 | Loss | *-* | *-* |
| 16q23.1 | Case | 75,239,764 | 75,356,481 | 116,718 |  | Loss | *-* | *-* |
| 16q23.1 | Case | 75,823,175 | 76,042,758 | 219,584 |  | Loss | *ADAMTS18* | *-* |
| 16q24.3 | Case | 87,778,383 | 87,786,976 | 8,594 | 0.017 | Loss | *CDH15* | *CDH15* |
| 17p12 | Case | 15,727,298 | 15,734,394 | 7,097 |  | Loss | *-* | *-* |
| 17p13.2 | Case | 4,293,704 | 4,365,674 | 71,971 |  | Gain | *SPNS3, SPNS2* | *SPNS3, SPNS2* |
| 17p13.3 | Case | 3,271,560 | 3,403,485 | 131,926 |  | Gain | *SPATA22, OR1E2, ASPA, TRPV3, OR3A3* | *TRPV3, OR3A3* |
| 17p13.3 | Case | 3,502,173 | 3,574,961 | 72,789 |  | Loss | *TAX1BP3, TMEM93, P2RX5, GSG2, CTNS, ITGAE* | *GSG2, CTNS, ITGAE* |
| 17p13.3 | Case | 3,524,871 | 3,538,571 | 13,701 |  | Loss | *P2RX5* | *P2RX5* |
| 17q12 | Case | 29,896,966 | 29,900,820 | 3,855 |  | Loss | *-* | *-* |
| 17q12 | Case | 32,788,938 | 32,832,784 | 43,847 |  | Loss | *C17orf78, ACACA* | *ACACA* |
| 17q12 | Case | 33,401,655 | 33,406,282 | 4,628 |  | Loss | *-* | *-* |
| 17q21.32 | Case | 43,751,830 | 43,757,211 | 5,382 |  | Loss | *SKAP1* | *-* |
| 17q21.33 | Case | 46,967,199 | 46,982,134 | 14,936 |  | Loss | *-* | *-* |
| 17q21.33 | Case | 46,975,449 | 46,988,478 | 13,030 |  | Loss | *-* | *-* |
| 17q22 | Case | 54,465,099 | 54,563,703 | 98,605 | 0.017 | Loss | *SKA2, TRIM37* | *SKA2, TRIM37* |
| 17q23.2 | Case | 55,767,143 | 55,808,507 | 41,365 |  | Loss | *USP32* | *USP32* |
| 17q25.1 | Case | 71,873,859 | 71,893,125 | 19,267 |  | Loss | *SPHK1* | *SPHK1* |
| 18p11.21 | Case | 14,270,824 | 14,274,131 | 3,308 |  | Loss | *-* | *-* |
| 18q11.2 | Case | 22,018,233 | 22,051,851 | 33,619 |  | Loss | *PSMA8* | *PSMA8* |
| 18q21.2 | Case | 49,390,468 | 49,392,954 | 2,487 |  | Loss | *-* | *-* |
| 18q22.3 | Case | 68,140,116 | 68,160,003 | 19,888 |  | Loss | *-* | *-* |
| 18q22.3 | Case | 70,023,001 | 70,588,212 | 565,212 |  | Gain | *CYB5A, FAM69C, LOC400657, ZNF407, CNDP1, CNDP2* | *ZNF407* |
| 18q23 | Case | 75,001,399 | 75,007,696 | 6,298 |  | Gain | *ATP9B* | *ATP9B* |
| 18q23 | Case | 75,410,988 | 75,414,447 | 3,460 |  | Loss | *-* | *-* |
| 19p13.11 | Case | 18,607,522 | 18,626,572 | 19,051 |  | Loss | *KLHL26* | *KLHL26* |
| 19p13.11 | Case | 18,607,522 | 18,641,799 | 34,278 |  | Loss | *KLHL26* | *KLHL26* |
| 19p13.3 | Case | 2,614,849 | 2,659,337 | 44,489 |  | Loss | *GNG7* | *GNG7* |
| 19q12 | Case | 33,882,358 | 33,934,258 | 51,901 |  | Loss | *-* | *-* |
| 19q13.33, 19q13.41 | Case | 57,492,119 | 57,619,851 | 127,733 |  | Loss | *ZNF480, ZNF610, ZNF528, NF880* | *-* |
| 20p12.3 | Case | 5,226,869 | 5,240,086 | 13,218 |  | Loss | *PROKR2* | *PROKR2* |
| 20p12.3 | Case | 7,635,324 | 7,652,428 | 17,105 |  | Loss | *-* | *-* |
| 20p12.3 | Case | 7,648,480 | 7,652,428 | 3,949 |  | Loss | *-* | *-* |
| 20q13.2 | Case | 54,290,616 | 54,296,092 | 5,477 | 0.026 | Loss | *-* | *-* |
| 21q22.3 | Case | 41,763,480 | 41,769,378 | 5,899 |  | Loss | *TMPRSS2* | *TMPRSS2* |
| 21q22.3 | Case | 43,121,107 | 43,219,454 | 98,348 |  | Gain | *NDUFV3, WDR4* | *-* |
| 21q22.3 | Case | 43,555,418 | 43,615,770 | 60,353 |  | Loss | *-* | *-* |
| 21q22.3 | Case | 45,255,220 | 45,291,102 | 35,883 |  | Loss | *-* | *-* |
| 22q13.31 | Case | 45,510,714 | 45,535,803 | 25,090 |  | Gain | *CERK* | *CERK* |
| 1p13.3 | Control | 108,493,305 | 108,531,282 | 37,978 |  | Loss | *SLC25A24* | *SLC25A24* |
| 1p21.1 | Control | 105,468,445 | 105,469,921 | 1,477 | 0.026 | Loss | *MIR548H3* | *-* |
| 1p21.1 | Control | 105,468,445 | 105,471,866 | 3,422 |  | Loss | *MIR548H3* | *-* |
| 1p34.3 | Control | 34,879,856 | 34,890,727 | 10,872 |  | Loss | *-* | *-* |
| 1p35.1 | Control | 32,294,381 | 32,320,443 | 26,063 |  | Loss | *TMEM39B* | *TMEM39B* |
| 1p36.21 | Control | 15,874,175 | 15,883,285 | 9,111 |  | Loss | *-* | *-* |
| 1p36.31 | Control | 5,986,242 | 5,989,223 | 2,982 |  | Loss | *-* | *-* |
| 1q23.2 | Control | 157,531,686 | 157,559,474 | 27,789 |  | Gain | *FCER1A, OR10J3* | *FCER1A* |
| 1q25.3 | Control | 183,253,348 | 183,279,972 | 26,625 |  | Loss | *-* | *-* |
| 1q31.1 | Control | 186,815,628 | 186,841,530 | 25,903 |  | Loss | *-* | *-* |
| 1q31.3 | Control | 197,018,024 | 197,041,764 | 23,741 |  | Loss | *-* | *-* |
| 1q31.3 | Control | 197,023,984 | 197,067,730 | 43,747 |  | Loss | *-* | *-* |
| 1q32.2 | Control | 205,603,594 | 205,612,121 | 8,528 |  | Loss | *-* | *-* |
| 1q32.2 | Control | 208,777,370 | 208,791,402 | 14,033 |  | Loss | *HHAT* | *-* |
| 1q42.13 | Control | 225,764,004 | 225,901,023 | 137,020 |  | Gain | *ZNF678* | *ZNF678* |
| 1q44 | Control | 244,747,739 | 244,759,678 | 11,940 |  | Loss | *-* | *-* |
| 2p11.2 | Control | 88,941,152 | 89,272,890 | 331,739 |  | Gain | *-* | *-* |
| 2p16.3 | Control | 48,692,459 | 48,711,100 | 18,642 | 0.017 | Loss | *GTF2A1L, STON1-GTF2A1L* | *GTF2A1L, STON1-GTF2A1L* |
| 2p16.3 | Control | 52,506,530 | 52,545,105 | 38,576 |  | Loss | *-* | *-* |
| 2p24.1 | Control | 19,789,116 | 19,806,295 | 17,180 |  | Loss | *-* | *-* |
| 2p24.1 | Control | 21,273,863 | 21,577,348 | 303,486 |  | Loss | *-* | *-* |
| 2p25.1 | Control | 11,728,737 | 11,772,547 | 43,811 |  | Loss | *-* | *-* |
| 2q12.3 | Control | 107,628,503 | 107,644,607 | 16,105 |  | Loss | *-* | *-* |
| 2q14.3 | Control | 122,284,084 | 122,553,663 | 269,580 |  | Loss | *-* | *-* |
| 2q22.1 | Control | 137,563,032 | 137,671,697 | 108,666 |  | Loss | *THSD7B* | *THSD7B* |
| 2q22.2 | Control | 143,780,306 | 143,794,640 | 14,335 |  | Loss | *ARHGAP15* | *-* |
| 2q32.1 | Control | 186,737,354 | 186,763,306 | 25,953 |  | Loss | *-* | *-* |
| 2q33.1 | Control | 201,886,334 | 201,988,385 | 102,052 |  | Loss | *TRAK2, ALS2CR12* | *TRAK2, ALS2CR12* |
| 2q33.1 | Control | 201,886,334 | 202,031,201 | 144,868 |  | Loss | *TRAK2, ALS2CR12, STRADB* | *ALS2CR12, STRADB* |
| 2q33.1 | Control | 201,904,308 | 201,993,112 | 88,805 |  | Loss | *TRAK2, ALS2CR12* | *TRAK2, ALS2CR12* |
| 2q33.1 | Control | 202,603,408 | 202,608,635 | 5,228 |  | Gain | *FZD7* | *FZD7* |
| 2q33.2 | Control | 203,924,804 | 204,046,729 | 121,926 |  | Loss | *ABI2, RAPH1* | *ABI2, RAPH1* |
| 2q34 | Control | 212,705,269 | 212,859,475 | 154,207 |  | Gain | *ERBB4* | *-* |
| 2q35 | Control | 219,757,534 | 219,762,670 | 5,137 |  | Loss | *FAM134A* | *FAM134A* |
| 2q35 | Control | 220,263,055 | 220,301,758 | 38,704 |  | Loss | *-* | *-* |
| 3p13 | Control | 71,856,875 | 71,886,828 | 29,954 |  | Gain | *EIF4E3, GPR27* | *EIF4E3, GPR27* |
| 3p13 | Control | 72,863,716 | 72,895,436 | 31,721 |  | Loss | *SHQ1* | *SHQ1* |
| 3p14.1 | Control | 65,087,061 | 65,094,573 | 7,513 |  | Loss | *-* | *-* |
| 3p14.3 | Control | 56,572,994 | 56,591,794 | 18,801 |  | Loss | *CCDC66* | *CCDC66* |
| 3p21.31 | Control | 47,907,278 | 47,933,041 | 25,764 |  | Gain | *MAP4* | *MAP4* |
| 3p21.31 | Control | 47,961,214 | 48,337,754 | 376,541 |  | Gain | *NME6, CDC25A, CAMP, MAP4, SPINK8, ZNF589* | *MAP4, SPINK8* |
| 3p24.3 | Control | 15,820,217 | 15,829,047 | 8,831 |  | Loss | *ANKRD28* | *-* |
| 3p24.3 | Control | 20,870,746 | 20,873,948 | 3,203 |  | Loss | *-* | *-* |
| 3p24.3 | Control | 22,683,806 | 22,716,468 | 32,663 |  | Loss | *-* | *-* |
| 3p24.3 | Control | 22,692,880 | 22,699,346 | 6,467 |  | Loss | *-* | *-* |
| 3q11.2 | Control | 96,938,051 | 96,950,842 | 12,792 |  | Gain | *-* | *-* |
| 3q11.2 | Control | 96,948,623 | 96,975,161 | 26,539 |  | Gain | *-* | *-* |
| 3q12.1 | Control | 101,403,574 | 101,437,411 | 33,838 |  | Loss | *-* | *-* |
| 3q13.11 | Control | 105,517,723 | 105,798,404 | 280,682 |  | Loss | *-* | *-* |
| 3q13.31 | Control | 118,118,878 | 118,353,538 | 234,661 |  | Loss | *-* | *-* |
| 3q13.32 | Control | 120,225,538 | 120,294,717 | 69,180 |  | Gain | *IGSF11* | *IGSF11* |
| 3q21.2 | Control | 126,391,868 | 126,420,793 | 28,926 |  | Loss | *SLC12A8* | *SLC12A8* |
| 3q24 | Control | 146,470,656 | 146,524,529 | 53,874 |  | Loss | *-* | *-* |
| 3q24 | Control | 147,846,875 | 147,867,771 | 20,897 |  | Loss | *-* | *-* |
| 3q26.1 | Control | 165,529,195 | 165,584,968 | 55,774 |  | Gain | *-* | *-* |
| 3q26.1 | Control | 168,327,715 | 168,351,902 | 24,188 | 0.017 | Loss | *-* | *-* |
| 3q27.3 | Control | 187,575,268 | 187,678,017 | 102,750 |  | Gain | *-* | *-* |
| 3q29 | Control | 197,672,005 | 197,796,521 | 124,517 |  | Loss | *FBXO45, RNF168, WDR53, C3orf43* | *FBXO45* |
| 4p15.2 | Control | 25,868,467 | 25,903,984 | 35,518 |  | Loss | *-* | *-* |
| 4p15.2 | Control | 25,883,008 | 25,903,984 | 20,977 |  | Loss | *-* | *-* |
| 4p16.1 | Control | 7,486,622 | 7,495,078 | 8,457 |  | Loss | *SORCS2, PSAPL1* | *PSAPL1* |
| 4p16.2 | Control | 3,731,921 | 4,031,034 | 299,114 |  | Gain | *LOC348926, ADRA2C* | *-* |
| 4q21.1 | Control | 77,173,431 | 77,193,375 | 19,945 |  | Loss | *CXCL11, ART3* | *-* |
| 4q28.2 | Control | 129,201,535 | 129,232,088 | 30,554 |  | Gain | *LARP1B* | *LARP1B* |
| 4q28.2 | Control | 130,349,762 | 130,365,626 | 15,865 |  | Loss | *-* | *-* |
| 4q31.3 | Control | 152,428,570 | 152,463,768 | 35,199 |  | Loss | *PRSS48* | *PRSS48* |
| 4q31.3 | Control | 153,221,112 | 153,279,667 | 58,556 |  | Loss | *-* | *-* |
| 5p13.2 | Control | 37,875,375 | 37,876,399 | 1,025 | 0.017 | Gain | *GDNF* | *GDNF* |
| 5p13.3 | Control | 29,363,668 | 29,604,932 | 241,265 |  | Gain | *-* | *-* |
| 5p13.3 | Control | 31,388,333 | 31,390,545 | 2,213 |  | Loss | *-* | *-* |
| 5p14.1 | Control | 25,855,082 | 25,859,429 | 4,348 |  | Loss | *-* | *-* |
| 5p14.1 | Control | 26,833,538 | 26,852,428 | 18,891 |  | Loss | *-* | *-* |
| 5p14.1 | Control | 28,682,174 | 28,755,438 | 73,265 |  | Loss | *-* | *-* |
| 5p14.1 | Control | 28,782,832 | 28,968,638 | 185,807 |  | Loss | *-* | *-* |
| 5p14.3 | Control | 20,868,373 | 20,927,962 | 59,590 |  | Loss | *-* | *-* |
| 5p14.3 | Control | 21,475,697 | 21,488,174 | 12,478 | 0.017 | Gain | *-* | *-* |
| 5p15.33 | Control | 1,223,732 | 1,233,567 | 9,836 |  | Loss | *-* | *-* |
| 5q14.2 | Control | 81,422,512 | 81,463,804 | 41,293 |  | Loss | *ATG10* | *-* |
| 5q21.1 | Control | 99,226,818 | 99,257,197 | 30,380 |  | Loss | *-* | *-* |
| 5q21.1 | Control | 101,725,362 | 101,823,389 | 98,028 |  | Gain | *SLCO6A1* | *SLCO6A1* |
| 5q22.3 | Control | 114,330,486 | 114,359,532 | 29,047 |  | Loss | *-* | *-* |
| 5q31.1 | Control | 135,117,551 | 135,148,416 | 30,866 |  | Gain | *-* | *-* |
| 5q31.1 | Control | 135,143,450 | 135,174,222 | 30,773 |  | Loss | *-* | *-* |
| 5q33.3 | Control | 158,828,019 | 158,833,594 | 5,576 |  | Loss | *-* | *-* |
| 5q34 | Control | 160,128,883 | 160,134,694 | 5,812 | 0.017 | Loss | *ATP10B* | *-* |
| 5q35.3 | Control | 178,447,335 | 178,449,089 | 1,755 |  | Loss | *-* | *-* |
| 6p21.31 | Control | 33,695,421 | 33,722,105 | 26,685 | 0.017 | Loss | *ITPR3* | *ITPR3* |
| 6p21.32 | Control | 33,157,957 | 33,162,024 | 4,068 |  | Loss | *HLA-DPB1* | *HLA-DPB1* |
| 6p21.33 | Control | 30,467,676 | 30,505,398 | 37,723 |  | Loss | *-* | *-* |
| 6p22.2 | Control | 24,433,606 | 24,437,709 | 4,104 |  | Loss | *DCDC2* | *-* |
| 6p22.2 | Control | 25,558,937 | 25,562,994 | 4,058 | 0.017 | Loss | *LRRC16A* | *-* |
| 6p22.2 | Control | 25,559,638 | 25,562,994 | 3,357 |  | Loss | *LRRC16A* | *-* |
| 6p22.3 | Control | 22,160,776 | 22,164,110 | 3,335 |  | Loss | *FLJ22536* | *-* |
| 6q12 | Control | 64,647,424 | 64,716,611 | 69,188 |  | Loss | *EYS* | *-* |
| 6q12 | Control | 64,725,004 | 64,736,715 | 11,712 |  | Loss | *EYS* | *-* |
| 6q16.1 | Control | 95,250,144 | 95,348,547 | 98,404 |  | Loss | *-* | *-* |
| 6q21 | Control | 108,717,334 | 108,720,595 | 3,262 |  | Loss | *-* | *-* |
| 6q24.3 | Control | 145,747,167 | 145,751,064 | 3,898 |  | Loss | *-* | *-* |
| 6q25.2 | Control | 153,974,143 | 154,005,890 | 31,748 |  | Loss | *-* | *-* |
| 7p15.1 | Control | 31,201,400 | 31,285,947 | 84,548 |  | Loss | *-* | *-* |
| 7p15.1 | Control | 31,556,844 | 31,560,691 | 3,848 |  | Loss | *CCDC129* | *CCDC129* |
| 7p15.1 | Control | 31,556,844 | 31,598,119 | 41,276 |  | Loss | *CCDC129* | *CCDC129* |
| 7p15.3 | Control | 20,711,068 | 20,719,998 | 8,931 | 0.017 | Loss | *ABCB5* | *-* |
| 7p15.3 | Control | 23,980,258 | 24,018,968 | 38,711 | 0.017 | Loss | *-* | *-* |
| 7p15.3 | Control | 24,011,759 | 24,021,536 | 9,778 |  | Loss | *-* | *-* |
| 7p21.3 | Control | 10,309,563 | 10,338,709 | 29,147 |  | Loss | *-* | *-* |
| 7p21.3 | Control | 12,137,021 | 12,187,286 | 50,266 |  | Loss | *-* | *-* |
| 7q31.1 | Control | 113,608,607 | 113,650,620 | 42,014 |  | Gain | *-* | *-* |
| 7q36.3 | Control | 157,128,182 | 157,155,841 | 27,660 |  | Loss | *PTPRN2* | *PTPRN2* |
| 8p12 | Control | 29,956,134 | 29,957,787 | 1,654 |  | Loss | *-* | *-* |
| 8p21.2 | Control | 25,124,828 | 25,157,853 | 33,026 |  | Gain | *DOCK5* | *DOCK5* |
| 8p23.2 | Control | 3,165,756 | 3,169,776 | 4,021 |  | Loss | *CSMD1* | *-* |
| 8q13.2 | Control | 69,055,794 | 69,067,529 | 11,736 |  | Loss | *PREX2* | *-* |
| 8q21.12 | Control | 80,019,495 | 80,022,801 | 3,307 |  | Loss | *-* | *-* |
| 8q22.1 | Control | 97,588,101 | 97,590,565 | 2,465 |  | Loss | *SDC2* | *-* |
| 8q23.3 | Control | 112,328,013 | 112,359,916 | 31,904 |  | Loss | *-* | *-* |
| 8q24.21 | Control | 131,439,413 | 131,461,056 | 21,644 |  | Gain | *ASAP1* | *ASAP1* |
| 8q24.22 | Control | 131,739,777 | 131,843,359 | 103,583 |  | Gain | *-* | *-* |
| 8q24.22 | Control | 131,868,477 | 131,911,818 | 43,342 |  | Gain | *ADCY8* | *ADCY8* |
| 8q24.22 | Control | 131,929,989 | 132,042,948 | 112,960 |  | Gain | *ADCY8* | *ADCY8* |
| 8q24.23 | Control | 138,790,588 | 138,812,960 | 22,373 | 0.017 | Loss | *-* | *-* |
| 8q24.3 | Control | 142,883,123 | 142,902,704 | 19,582 |  | Loss | *-* | *-* |
| 8q24.3 | Control | 143,643,001 | 143,760,960 | 117,960 |  | Loss | *ARC, JRK, PSCA* | *PSCA* |
| 9p21.1 | Control | 30,964,923 | 31,282,431 | 317,509 |  | Gain | *-* | *-* |
| 9q22.1 | Control | 89,760,155 | 89,859,123 | 98,969 |  | Gain | *CDK20* | *-* |
| 9q22.1 | Control | 89,902,132 | 89,905,289 | 3,158 |  | Loss | *-* | *-* |
| 9q22.32 | Control | 96,638,787 | 97,359,032 | 720,246 |  | Gain | *MIR27B, MIR23B, FANCC, C9orf3, PTCH1, MIR24-1* | *C9orf3* |
| 9q31.1 | Control | 105,329,248 | 105,644,054 | 314,807 |  | Gain | *-* | *-* |
| 9q34.11 | Control | 130,452,370 | 130,605,375 | 153,006 |  | Gain | *SET, ZDHHC12, PKN3, ZER1, WDR34, TBC1D13* | *WDR34, TBC1D13* |
| 9q34.11 | Control | 130,960,002 | 131,036,783 | 76,782 |  | Gain | *IER5L* | *-* |
| 9q34.11 | Control | 131,255,364 | 131,348,664 | 93,301 |  | Loss | *-* | *-* |
| 9q34.12 | Control | 132,539,954 | 132,636,413 | 96,460 |  | Gain | *EXOSC2, ABL1, PRDM12* | *ABL1, PRDM12* |
| 9q34.13 | Control | 132,925,525 | 133,213,012 | 287,488 |  | Gain | *NUP214, LAMC3, AIF1L, FAM78A, PPAPDC3* | *LAMC3* |
| 10p12.1 | Control | 27,037,924 | 27,041,820 | 3,897 | 0.017 | Loss | *PDSS1* | *PDSS1* |
| 10p12.31 | Control | 20,687,518 | 20,696,836 | 9,319 |  | Loss | *-* | *-* |
| 10p12.33 | Control | 19,315,664 | 19,491,734 | 176,071 |  | Loss | *-* | *-* |
| 10p14 | Control | 8,029,055 | 8,036,814 | 7,760 | 0.017 | Loss | *TAF3* | *-* |
| 10p14 | Control | 8,029,055 | 8,049,159 | 20,105 | 0.017 | Loss | *TAF3* | *TAF3* |
| 10q21.1 | Control | 57,769,091 | 57,922,796 | 153,706 |  | Gain | *ZWINT* | *-* |
| 10q21.3 | Control | 70,845,615 | 70,972,433 | 126,819 |  | Gain | *TACR2, TSPAN15* | *TACR2* |
| 10q26.3 | Control | 132,721,809 | 132,731,220 | 9,412 |  | Loss | *-* | *-* |
| 10q26.3 | Control | 133,070,596 | 133,094,517 | 23,922 |  | Loss | *-* | *-* |
| 10q26.3 | Control | 134,763,348 | 134,818,454 | 55,107 |  | Gain | *GPR123* | *GPR123* |
| 11p12 | Control | 38,278,782 | 38,317,414 | 38,633 |  | Loss | *-* | *-* |
| 11p14.1 | Control | 28,963,954 | 28,993,215 | 29,262 | 0.017 | Gain | *-* | *-* |
| 11p14.1 | Control | 29,921,416 | 29,924,814 | 3,399 |  | Loss | *-* | *-* |
| 11p14.3 | Control | 21,796,469 | 21,805,901 | 9,433 | 0.017 | Loss | *-* | *-* |
| 11p14.3 | Control | 24,231,908 | 24,247,394 | 15,487 |  | Loss | *-* | *-* |
| 11p15.1 | Control | 18,925,482 | 18,977,997 | 52,516 |  | Loss | *-* | *-* |
| 11p15.1 | Control | 18,929,754 | 18,977,997 | 48,244 |  | Loss | *-* | *-* |
| 11p15.3 | Control | 11,832,323 | 11,846,995 | 14,673 |  | Loss | *USP47* | *-* |
| 11p15.4 | Control | 6,622,630 | 6,634,798 | 12,169 |  | Loss | *DCHS1* | *DCHS1* |
| 11p15.5 | Control | 1,386,192 | 1,390,272 | 4,081 |  | Gain | *BRSK2* | *-* |
| 11p15.5 | Control | 1,611,186 | 1,619,986 | 8,801 |  | Loss | *MOB2* | *-* |
| 11q21 | Control | 96,564,127 | 96,644,619 | 80,493 |  | Gain | *-* | *-* |
| 11q22.3 | Control | 103,743,297 | 103,843,550 | 100,254 |  | Loss | *-* | *-* |
| 11q22.3 | Control | 106,291,638 | 106,311,230 | 19,593 |  | Loss | *GUCY1A2* | *-* |
| 11q22.3 | Control | 106,697,467 | 106,756,061 | 58,595 | 0.017 | Loss | *CWF19L2* | *CWF19L2* |
| 12q15 | Control | 68,966,131 | 68,972,330 | 6,200 |  | Loss | *CNOT2* | *-* |
| 13q12.13 | Control | 25,948,284 | 25,952,921 | 4,638 |  | Loss | *-* | *-* |
| 13q14.2 | Control | 47,152,997 | 47,195,839 | 42,843 |  | Loss | *-* | *-* |
| 13q21.1 | Control | 52,318,345 | 52,321,106 | 2,762 |  | Gain | *PCDH8* | *PCDH8* |
| 13q21.33 | Control | 69,162,578 | 69,393,269 | 230,692 |  | Gain | *KLHL1* | *KLHL1* |
| 13q21.33 | Control | 71,743,964 | 71,746,817 | 2,854 |  | Loss | *-* | *-* |
| 13q32.1 | Control | 96,894,883 | 96,906,362 | 11,480 |  | Loss | *RAP2A* | *-* |
| 13q32.2 | Control | 98,049,901 | 98,064,782 | 14,882 |  | Loss | *-* | *-* |
| 13q32.2 | Control | 98,049,901 | 98,070,051 | 20,151 |  | Loss | *-* | *-* |
| 13q34 | Control | 110,364,577 | 110,369,322 | 4,746 | 0.017 | Gain | *ANKRD10* | *ANKRD10* |
| 13q34 | Control | 112,535,044 | 112,550,789 | 15,746 |  | Loss | *ATP11A* | *ATP11A* |
| 13q34 | Control | 113,938,930 | 113,954,191 | 15,262 |  | Loss | *-* | *-* |
| 14q23.2 | Control | 63,318,302 | 63,358,915 | 40,614 |  | Loss | *-* | *-* |
| 14q32.13 | Control | 93,514,235 | 93,534,242 | 20,008 |  | Loss | *C14orf48* | *C14orf48* |
| 14q32.31 | Control | 100,991,643 | 100,998,138 | 6,496 | 0.017 | Gain | *-* | *-* |
| 14q32.33 | Control | 105,275,606 | 105,599,826 | 324,221 |  | Gain | *ADAM6, KIAA0125* | *-* |
| 14q32.33 | Control | 105,307,385 | 105,638,853 | 331,469 |  | Gain | *ADAM6, KIAA0125* | *-* |
| 14q32.33 | Control | 105,461,641 | 105,786,123 | 324,483 |  | Gain | *ADAM6, KIAA0125* | *KIAA0125* |
| 14q32.33 | Control | 105,522,072 | 105,852,187 | 330,116 |  | Gain | *-* | *-* |
| 15q12 | Control | 24,279,967 | 24,303,361 | 23,395 | 0.026 | Loss | *-* | *-* |
| 15q21.1 | Control | 47,327,508 | 47,335,082 | 7,575 |  | Loss | *GALK2* | *-* |
| 15q21.1 | Control | 47,332,301 | 47,337,235 | 4,935 |  | Loss | *GALK2* | *-* |
| 15q22.31 | Control | 63,603,964 | 63,607,883 | 3,920 | 0.035 | Loss | *-* | *-* |
| 15q26.2 | Control | 92,687,437 | 92,703,955 | 16,519 |  | Gain | *MCTP2* | *MCTP2* |
| 15q26.2 | Control | 93,844,426 | 93,850,281 | 5,856 |  | Loss | *LOC145820* | *LOC145820* |
| 15q26.3 | Control | 99,145,509 | 99,159,827 | 14,319 | 0.017 | Loss | *-* | *-* |
| 16p11.2 | Control | 28,447,349 | 28,539,086 | 91,738 |  | Loss | *CCDC101, NUPR1, SULT1A1, SULT1A2* | *SULT1A1* |
| 16p12.1 | Control | 22,953,373 | 22,956,947 | 3,575 | 0.017 | Loss | *-* | *-* |
| 16p12.1 | Control | 22,955,734 | 22,958,527 | 2,794 |  | Loss | *-* | *-* |
| 16p12.3 | Control | 16,767,302 | 17,042,950 | 275,649 |  | Gain | *-* | *-* |
| 16q13 | Control | 55,923,777 | 55,944,067 | 20,291 |  | Loss | *-* | *-* |
| 16q13 | Control | 55,923,879 | 55,932,768 | 8,890 | 0.017 | Loss | *-* | *-* |
| 16q21 | Control | 61,101,275 | 61,111,714 | 10,440 |  | Loss | *-* | *-* |
| 16q21 | Control | 61,101,966 | 61,116,069 | 14,104 |  | Loss | *-* | *-* |
| 16q22.3 | Control | 70,983,949 | 71,193,175 | 209,227 |  | Loss | *-* | *-* |
| 16q24.2 | Control | 87,095,378 | 87,166,355 | 70,978 |  | Gain | *ZC3H18, ZFPM1* | *ZC3H18, ZFPM1* |
| 16q24.3 | Control | 87,677,415 | 87,788,064 | 110,650 |  | Loss | *C16orf81, ACSF3, CDH15* | *CDH15* |
| 16q24.3 | Control | 88,425,222 | 88,506,995 | 81,774 |  | Loss | *TCF25, SPIRE2* | *SPIRE2* |
| 17p13.2 | Control | 5,616,762 | 5,618,001 | 1,240 |  | Loss | *-* | *-* |
| 17p13.2 | Control | 5,683,518 | 5,745,734 | 62,217 |  | Gain | *-* | *-* |
| 17p13.3 | Control | 357,165 | 359,589 | 2,425 |  | Loss | *-* | *-* |
| 17p13.3 | Control | 3,267,156 | 3,378,436 | 111,281 |  | Gain | *SPATA22, OR1E2, ASPA, TRPV3, OR3A3* | *TRPV3* |
| 17p13.3 | Control | 3,535,733 | 3,547,057 | 11,325 |  | Loss | *P2RX5* | *P2RX5* |
| 17q11.2 | Control | 26,578,490 | 26,588,729 | 10,240 |  | Loss | *NF1* | *NF1* |
| 17q12 | Control | 29,896,966 | 29,913,091 | 16,126 |  | Loss | *-* | *-* |
| 17q21.32 | Control | 43,755,535 | 43,785,144 | 29,610 |  | Loss | *SKAP1* | *SKAP1* |
| 17q21.33 | Control | 44,875,052 | 44,965,370 | 90,319 |  | Loss | *NGFR* | *-* |
| 17q25.1 | Control | 71,884,974 | 71,893,787 | 8,814 |  | Gain | *SPHK1* | *SPHK1* |
| 18p11.21 | Control | 11,496,644 | 11,501,222 | 4,579 |  | Gain | *-* | *-* |
| 18p11.21 | Control | 12,647,195 | 12,663,574 | 16,380 | 0.044 | Gain | *CEP76, SPIRE1* | *CEP76, SPIRE1* |
| 18p11.31 | Control | 5,306,702 | 5,316,221 | 9,520 |  | Loss | *-* | *-* |
| 18p11.31 | Control | 5,313,289 | 5,322,222 | 8,934 |  | Loss | *-* | *-* |
| 18p11.31 | Control | 5,314,786 | 5,322,222 | 7,437 |  | Loss | *-* | *-* |
| 18q12.2 | Control | 33,560,099 | 33,603,347 | 43,249 |  | Loss | *-* | *-* |
| 18q12.3 | Control | 37,237,403 | 37,349,005 | 111,603 |  | Loss | *KC6* | *KC6* |
| 18q12.3 | Control | 38,910,529 | 39,006,193 | 95,665 |  | Gain | *RIT2* | *RIT2* |
| 18q12.3 | Control | 39,920,535 | 39,948,521 | 27,987 |  | Gain | *-* | *-* |
| 18q12.3 | Control | 41,544,541 | 41,571,280 | 26,740 |  | Gain | *SLC14A1* | *SLC14A1* |
| 18q21.1 | Control | 43,993,633 | 44,018,978 | 25,346 |  | Loss | *-* | *-* |
| 18q21.33 | Control | 57,856,162 | 57,862,290 | 6,129 |  | Gain | *-* | *-* |
| 18q22.3 | Control | 68,144,721 | 68,166,130 | 21,410 |  | Loss | *-* | *-* |
| 18q23 | Control | 75,141,310 | 75,159,977 | 18,668 |  | Loss | *ATP9B* | *-* |
| 18q23 | Control | 75,414,447 | 75,486,867 | 72,421 |  | Loss | *-* | *-* |
| 19p13.12 | Control | 13,953,009 | 14,063,151 | 110,143 |  | Gain | *PALM3, IL27RA, RFX1, LOC113230, SAMD1, RLN3* | *RFX1* |
| 19p13.2 | Control | 12,466,955 | 12,585,645 | 118,691 |  | Loss | *ZNF709, ZNF564, ZNF791, ZNF490* | *ZNF709, ZNF791* |
| 19p13.2 | Control | 12,525,147 | 12,558,389 | 33,243 |  | Loss | *ZNF490* | *ZNF490* |
| 19p13.3 | Control | 3,854,004 | 3,895,240 | 41,237 |  | Loss | *ATCAY, ITGB1BP3* | *ATCAY* |
| 19p13.3 | Control | 3,888,060 | 3,895,240 | 7,181 |  | Loss | *ITGB1BP3* | *ITGB1BP3* |
| 19q12 | Control | 32,848,506 | 33,005,128 | 156,623 |  | Loss | *LOC148189* | *-* |
| 19q12 | Control | 35,979,673 | 35,984,184 | 4,512 |  | Gain | *-* | *-* |
| 19q13.11 | Control | 37,899,485 | 37,942,530 | 43,046 |  | Loss | *TDRD12* | *TDRD12* |
| 19q13.2 | Control | 46,359,843 | 46,403,655 | 43,813 |  | Loss | *CYP2S1* | *CYP2S1* |
| 19q13.31 | Control | 48,961,471 | 49,000,842 | 39,372 |  | Gain | *KCNN4, LYPD5* | *LYPD5* |
| 19q13.31 | Control | 49,647,767 | 49,659,327 | 11,561 |  | Loss | *-* | *-* |
| 19q13.42 | Control | 59,247,905 | 59,304,440 | 56,536 |  | Loss | *OSCAR, TARM1, TFPT, VSTM1, NDUFA3* | *TFPT, VSTM1* |
| 19q13.42 | Control | 59,953,516 | 59,986,141 | 32,626 |  | Loss | *KIR2DL3, KIR2DL1, KIR3DP1* | *KIR2DL3, KIR2DL1, KIR3DP1* |
| 19q13.43 | Control | 63,740,123 | 63,754,071 | 13,949 |  | Loss | *TRIM28* | *-* |
| 20p12.3 | Control | 7,347,802 | 7,372,828 | 25,027 | 0.026 | Loss | *-* | *-* |
| 20p12.3 | Control | 7,607,991 | 7,652,428 | 44,438 |  | Loss | *-* | *-* |
| 20p12.3 | Control | 7,640,889 | 7,652,428 | 11,540 |  | Loss | *-* | *-* |
| 20q13.13 | Control | 46,535,406 | 46,536,643 | 1,238 |  | Loss | *-* | *-* |
| 20q13.33 | Control | 60,091,799 | 60,174,325 | 82,527 | 0.026 | Gain | *LSM14B, PSMA7, SS18L1* | *SS18L1* |
| 21q22.3 | Control | 41,767,819 | 41,770,705 | 2,887 | 0.017 | Loss | *TMPRSS2* | *TMPRSS2* |
| 21q22.3 | Control | 46,397,978 | 46,444,212 | 46,235 |  | Loss | *C21orf56, FTCD, LSS* | *FTCD, LSS* |
| 22q11.21 | Control | 18,745,898 | 18,889,376 | 143,479 |  | Gain | *PI4KAP1, RIMBP3* | *-* |
| 22q12.2 | Control | 28,624,744 | 28,667,120 | 42,377 |  | Loss | *MTMR3* | *-* |
| 22q13.31 | Control | 44,032,602 | 44,035,828 | 3,227 |  | Loss | *-* | *-* |
