# Supplementary material for: Rare Copy Number Variants Identified Suggest the Regulating Pathways in Hypertension-Related Left Ventricular Hypertrophy
Source: PLoS One. 2016 Mar 1;11(3):e0148755. doi: 10.1371/journal.pone.0148755 (PMC4773219; doi:10.1371/journal.pone.0148755)
Supplement: S3 Table — (DOC) [file pone.0148755.s003.doc]

**S3 Table. Gene ontology and pathway analyses identified in hypertension-related LVH**

| **Gene functional category** | **Entrez Genes** | **Fold enrichment** | **P value** | **N** |
| --- | --- | --- | --- | --- |
| **DAVID pathways:** |  |  |  |  |
| Translational Initiation  (GO:000643) | *RSP17, EIF3C, EIF3CL, EIF2B3* | 12.53 | 0.003 |  |
| Translational factor activity, nucleic acid binding  (GO:0008135) | *EIF3C, EIF3CL, EEFSEC, EIF2B3* | 5.41 | 0.037 |  |
| Ion binding  (GO:0043167) | *RPS6KA2, ATP9B, MUTYH, SUMF1, TESK2, MYO9A, PADI4, SPHK1, ZNF300, ZNF528, ZNF610, TRPV3, MCTP2, ACACA, ADAMTS18, ITGAE, KCNIP4, GUCY1A2, CNDP1, CNDP2, ZNF880, ZNF407, TOE1, GABRA5, ZSWIM6, PXDN, ASPA, ZNF480, CYB5A, CERK, FLG, BRSK2, ZSWIM5, MMACHC, ZBTB11, CDH15, FAT2, GSG2, TRIM37, USP32,* | 1.31 | 0.029 |  |
| Enzyme activator activity  (GO:0008047) | *IQGAP2, MYO9A, RGS12, MGST2, SRGAP2* | 2.77 | 0.039 |  |
| Sohingoid metabolic process  (GO:0046519) | *SPHK1, CERK, CLN3* |  | 0.039 |  |
| Activation of protein kinase C, G-protein coupled receptor signaling  (GO:0007205) | *SPHK1, F2R, CERK* |  | 0.026 |  |
| **Ingenuity pathway analysis:** |  |  |  |  |
|  |  |  |  |  |
| Tissue development | *MGAT5, F2R, SPHK1, IL27, TFEB, P2RX5, NPR2, SKAP1* |  | 1.04E-04 - 4.91E-02 |  |
| Cell-to-cell signaling & interaction | *MGAT5, IL27, F2R, MGAT5, SPHK1, F2R, ITGAE, ABCC5, GNG7, NPR2* |  | 3.43E-04 - 4.65E-02 |  |
| Lipid metabolism | *CLN3, CERK, AKR1A1, F2R, PROKR2, IRGM, ACACA, SKAP1* |  | 2.60E-03 - 4.65E-02 |  |
| Small molecule biochemistry | *CLN3, TFEB, AKR1A1, ASPA, PROKR2, CERK, ACACA, SPHK1, F2R, IRGM, GUCY1A2, UROD, CLN3, ABCC5, SPHK1, MGAT5, SKAP1* |  | 2.60E-03 - 4.65E-02 |  |
| Cell morphology | *MGAT5, ACACA, F2R, DIAPH3, GABRA5, SKAP1* |  | 3.02E-03 |  |
| Cellular compromise | *F2R, DIAPH3, ASPA* |  | 3.02E-03 |  |
| Organ morphology | *F2R, NUPR1* |  | 3.02E-03 |  |
| Tissue morphology | *VAV3, IQGAP2, SPHK1, F2R, IL27, PRDX1, RUVBL1, TAX1BP3, GUCY1A2, SKAP1* |  |  |  |
| Embryonic development | *ABCC5, IQGAP2* |  | 4.46E-03 - 3.50E-02 |  |
| **MetaCore pathway maps:** |  |  |  |  |
| Regulation of purine nucleotide metabolic process | *GNG7, PTPRN2, GUCY1A2, VAV3, IQGAP2, SRGAP2, RGS12, MYO9A* |  | 1.28E-06 |  |
| Regulation of neuron migration | *ZSWIM6, SRGAP2* |  | 5.00E-06 |  |
| Intracellular signal transduction | *DCDC2, TIP1, FGFR1, TESK2, SPHK1, GUCY1A2, IQGAP2, VAV3, PDRX1, RABL2A, RPS6KA2, PSMA8, SRGAP2, MCTP2, GSG2, PAR1, MYO9A, NUPR1* |  | 1.22E-05 |  |
| Negative regulation of renin secretion into blood stream | *PAR1* |  | 4.03E-05 |  |
| Hydrogen peroxide catabolic process | *PRDX1, PXDN* |  | 4.35E-05 |  |

* N, number of subjects with hypertensive-left ventricular hypertrophy contributing at least one gene to the gene functional category.
