# Supplementary material for: Rare Copy Number Variants Identified Suggest the Regulating Pathways in Hypertension-Related Left Ventricular Hypertrophy
Source: PLoS One. 2016 Mar 1;11(3):e0148755. doi: 10.1371/journal.pone.0148755 (PMC4773219; doi:10.1371/journal.pone.0148755)
Supplement: S4 Table — (DOC) [file pone.0148755.s004.doc]

**S4 Table. Top significant disease groups identified by Ingenuity (IPA) enrichment analysis**

| **Disease** | **EntrezGenes** | **P value** |
| --- | --- | --- |
| Inflammatory response / disease | *F2R, SPHK1, MGAT5, DMBT1, CTNS, HLA-DQB1, IRGM, CTNS, VAV3, ABCC5* | 3.43E-04 - 4.65E-02 |
| Respiratory disease | *MUTYH, PRDX1, SPHK1, SKAP1* | 2.87E-03 - 4.08E-02 |
| Connective tissue disorder | *F2R, SKAP1, GUCY1A2, PRDX1, SPHK1* | 5.56E-03 - 2.85E-02 |
| Skeletal and muscular disorders | *GUCY1A2, SKAP1, SPHK1, PRDX1, DIAPH3* | 5.56E-03 - 3.50E-02 |
