# Supplementary material for: Rare Copy Number Variants Identified Suggest the Regulating Pathways in Hypertension-Related Left Ventricular Hypertrophy
Source: PLoS One. 2016 Mar 1;11(3):e0148755. doi: 10.1371/journal.pone.0148755 (PMC4773219; doi:10.1371/journal.pone.0148755)
Supplement: S5 Table — (DOC) [file pone.0148755.s005.doc]

**S5 Table. Top significant tissue groups identified by GO MetaCore enrichment analysis**

| **Tissue** | **P value** | **EntrezGenes** |
| --- | --- | --- |
| Heart | 1.47E-57 | *CLN3, RPS6KA2, F2R, PGD, PHGDH, STAG3L4, TRIM37, GNG7, ABCC5, DMBT1, TAX1BP3, TESK2, ATXN2L, CTSE, NUPR1, FOXJ3, ZNF407, SRGAP2, APOB48R, CDH15, FGFR1, HLA-DQB1, UROD, SRGAP3, USP32, CERK, PXDN, TFEB, MMACHC, SPHK1, MUTYH, HECTD3, LHFP, BRSK2, TMEM135, ACACA, PTGDR, AKR1A1, TTC4, TMPRSS2, EMC6, OR3A3, GUCY1A2, ITIH5, MYO9A, CTNS, CNDP2, PTPRN2, FAM5C, C19orf10, CYB5A, SKAP1, ITGAE, VAV3, RGS12, TOE1, NASP, EIF2B3, MGST2, PRDX1, ZBTB11, TRAK2, RUVBL1, GABRA5, P2RX5, IQGAP2, ATP9B, ASPA* |
| Skeletal muscle | 1.64E-52 | *CLN3, RPS6KA2, F2R, PGD, PHGDH, STAG3L4, TRIM37, GNG7, ABCC5, DMBT1, TAX1BP3, TESK2, ATXN2L, CTSE, NUPR1, FOXJ3, ZNF407, APOB48R, CDH15, FGFR1, HLA-DQB1, UROD, USP32, CERK, PADI4, PXDN, TFEB, MMACHC, SPHK1, MUTYH, HECTD3, LHFP, BRSK2, TMEM135, ACACA, PTGDR, AKR1A1, TTC4, TMPRSS2, EMC6, OR3A3, GUCY1A2, ITIH5, MYO9A, CTNS, CNDP2, PTPRN2, C19orf10, CYB5A, SKAP1, ITGAE, VAV3, RGS12, TOE1, NASP, EIF2B3, MGST2, PRDX1, ZBTB11, TRAK2, HLA-DQB2, RUVBL1, P2RX5, IQGAP2, ATP9B, ASPA* |
| Smooth muscle | 7.72E-52 | *CLN3, RPS6KA2, F2R, PHGDH, STAG3L4, TRIM37, ABCC5, DMBT1, TAX1BP3, TESK2, ATXN2L, CTSE, NUPR1, FOXJ3, ZNF407, SRGAP2, APOB48R, CDH15, FGFR1, HLA-DQB1, UROD, SRGAP3, USP32, CERK, PADI4, PXDN, TFEB, SPHK1, WDR4, MUTYH, HECTD3, LHFP, BRSK2, TMEM135, ACACA, PTGDR, AKR1A1, TTC4, TMPRSS2, EMC6, OR3A3, ITIH5, MYO9A, CTNS, CNDP2, PTPRN2, C19orf10, CYB5A, SKAP1, ITGAE, VAV3, RGS12, TOE1, NASP, EIF2B3, MGST2, PRDX1, ZBTB11, TRAK2, HLA-DQB2, RUVBL1, GABRA5, P2RX5, IQGAP2, ATP9B* |
