# Supplementary material for: Rare Copy Number Variants Identified Suggest the Regulating Pathways in Hypertension-Related Left Ventricular Hypertrophy
Source: PLoS One. 2016 Mar 1;11(3):e0148755. doi: 10.1371/journal.pone.0148755 (PMC4773219; doi:10.1371/journal.pone.0148755)
Supplement: S6 Table — Chr, chromosome; hg18, human genome assembly 18 (March 2006). Dashes indicate that no gene is involved or disrupted by CNV breakpoints. Highlighted are genes that are identified in the earlier stage of the study. (DOC) [file pone.0148755.s006.doc]

**S6 Table. Case- and control specific CNVs identified in the 116 hypertension related LVH subjects in the replication study.**

**Chr, chromosome; hg18, human genome assembly 18 (March 2006). Dashes indicate that no gene is involved or disrupted by CNV breakpoints. Highlighted are genes that are identified in the earlier stage of the study.**

| **Chr Cytoband** | **Case/ control** | **Start** | **End** | **Size** | **CNV type**  **(gain/loss)** | **Genes involved** | **Disrupted genes** |
| --- | --- | --- | --- | --- | --- | --- | --- |
| 10p11.21 | case | 34,731,264 | 34,821,063 | 89,800 | Gain | *PARD3* | *PARD3* |
| 10p12.31 | case | 19,272,870 | 19,451,728 | 178,859 | Loss | *-* | *-* |
| 10p14 | case | 6,677,767 | 6,835,673 | 157,907 | Loss | *-* | *-* |
| 10q21.1 | case | 53,114,079 | 53,218,886 | 104,808 | Gain | *PRKG1* | *-* |
| 10q21.1 | case | 58,919,271 | 59,079,675 | 160,405 | Gain | *-* | *-* |
| 10q25.3 | case | 116,354,196 | 117,104,965 | 750,770 | Gain | *FAM160B1,TRUB1,ABLIM1,ATRNL1* | *FAM160B1,TRUB1,ABLIM1,ATRNL1* |
| 10q26.13 | case | 126,054,225 | 126,102,896 | 48,672 | Gain | *OAT* | *OAT* |
| 10q26.2 | case | 130,445,346 | 130,466,658 | 21,313 | Gain | *-* | *-* |
| 11p13 | case | 35,244,058 | 35,257,316 | 13,259 | Gain | *CD44* | *CD44* |
| 11p15.3 | case | 12,166,233 | 12,172,427 | 6,195 | Loss | *MICAL2* | *-* |
| 11p15.5 | case | 290,158 | 316,299 | 26,142 | Loss | *IFITM2,IFITM1,ATHL1,IFITM5* | *IFITM2,IFITM1,ATHL1,IFITM5* |
| 12p11.23 | case | 27,315,291 | 27,788,795 | 473,505 | Gain | *SMCO2,ARNTL2,STK38L,PPFIBP1* | *SMCO2,ARNTL2,STK38L,PPFIBP1* |
| 12q23.1 | case | 100,079,988 | 100,087,408 | 7,421 | Loss | *ANKS1B* | *-* |
| 12q23.1 | case | 100,079,988 | 100,087,408 | 7,421 | Loss | *ANKS1B* | *-* |
| 13q12.11 | case | 22,155,903 | 22,239,305 | 83,403 | Gain | *MICU2* | *MICU2* |
| 13q21.33 | case | 69,633,096 | 69,696,093 | 62,998 | Loss | *-* | *-* |
| 13q31.1 | case | 86,590,651 | 86,594,356 | 3,706 | Loss | *-* | *-* |
| 13q31.3 | case | 91,441,129 | 91,498,214 | 57,086 | Gain | *-* | *-* |
| 14q13.1 | case | 33,331,841 | 33,345,632 | 13,792 | Gain | *-* | *-* |
| 14q13.1 | case | 33,651,895 | 33,676,728 | 24,834 | Loss | *NPAS3* | *-* |
| 14q23.3 | case | 65,996,619 | 66,597,985 | 601,367 | Gain | *FUT8* | *FUT8* |
| 15q21.1 | case | 45,456,194 | 45,464,768 | 8,575 | Gain | *DUOX1,SHF* | *DUOX1,SHF* |
| 15q22.31 | case | 66,438,551 | 66,452,847 | 14,297 | Loss | *MEGF11* | *-* |
| 15q26.3 | case | 99,127,913 | 99,131,822 | 3,910 | Gain | *-* | *-* |
| 15q26.3 | case | 101,200,141 | 101,329,941 | 129,801 | Gain | *-* | *-* |
| 16p12.1 | case | 27,075,427 | 27,080,306 | 4,880 | Gain | *C16orf82* | *C16orf82* |
| 16p13.2 | case | 9,417,781 | 9,438,692 | 20,912 | Gain | *-* | *-* |
| 16p13.2 | case | 9,474,056 | 9,528,277 | 54,222 | Gain | *-* | *-* |
| 16q21 | case | 63,430,369 | 63,473,919 | 43,551 | Loss | *-* | *-* |
| 16q24.3 | case | 89,094,243 | 89,185,019 | 90,777 | Gain | *ACSF3* | *ACSF3* |
| 17p13.2 | case | 5,742,794 | 5,773,602 | 30,809 | Gain | *-* | *-* |
| 17p13.2 | case | 5,742,794 | 5,773,602 | 30,809 | Gain | *-* | *-* |
| 18p11.23,  18p11.31 | case | 6,873,354 | 7,433,519 | 560,166 | Gain | *LRRC30,LAMA1,ARHGAP28* | *LRRC30,LAMA1,ARHGAP28* |
| 19p13.11 | case | 19,882,476 | 19,939,387 | 56,912 | Gain | *ZNF506* | *ZNF506* |
| 1p13.3 | case | 108,181,947 | 108,190,581 | 8,635 | Gain | ***VAV3*** | *VAV3* |
| 1p13.3 | case | 108,596,968 | 108,609,194 | 12,227 | Loss | *-* | *-* |
| 1p31.1 | case | 71,360,150 | 71,361,326 | 1,177 | Loss | *PTGER3* | *-* |
| 1p31.1 | case | 71,360,150 | 71,361,673 | 1,524 | Loss | *PTGER3* | *-* |
| 1q21.3 | case | 151,514,603 | 151,653,312 | 138,710 | Gain | *TUFT1,SNX27* | *TUFT1,SNX27* |
| 1q25.3 | case | 184,824,457 | 184,967,014 | 142,558 | Gain | *FAM129A* | *FAM129A* |
| 1q31.1 | case | 186,377,439 | 186,447,591 | 70,153 | Gain | *C1orf27,PDC* | *C1orf27,PDC* |
| 1q32.2 | case | 207,955,823 | 207,968,257 | 12,435 | Gain | *CD46* | *CD46* |
| 1q32.3 | case | 213,003,428 | 213,020,221 | 16,794 | Loss | *C1orf227* | *C1orf227* |
| 1q44 | case | 244,422,816 | 244,483,375 | 60,560 | Gain | *-* | *-* |
| 20q11.21 | case | 29,889,768 | 29,995,517 | 105,750 | Gain | *DEFB116,DEFB121,DEFB118,DEFB119* | *DEFB116,DEFB121,DEFB118,DEFB119* |
| 21q21.1 | case | 18,720,293 | 18,723,671 | 3,379 | Loss | *-* | *-* |
| 21q21.1 | case | 18,720,293 | 18,723,671 | 3,379 | Loss | *-* | *-* |
| 21q21.1 | case | 18,720,293 | 18,723,671 | 3,379 | Loss | *-* | *-* |
| 21q21.1 | case | 18,720,293 | 18,723,671 | 3,379 | Loss | *-* | *-* |
| 21q21.2 | case | 24,819,148 | 24,832,028 | 12,881 | Loss | *-* | *-* |
| 21q21.2 | case | 24,819,148 | 24,832,028 | 12,881 | Loss | *-* | *-* |
| 21q22.11 | case | 34,601,021 | 34,602,321 | 1,301 | Gain | *IFNAR2* | *-* |
| 21q22.11 | case | 34,601,021 | 34,602,321 | 1,301 | Gain | *IFNAR2* | *-* |
| 2p13.2 | case | 72,251,751 | 72,273,058 | 21,308 | Loss | *-* | *-* |
| 2p13.2 | case | 72,254,126 | 72,273,058 | 18,933 | Loss | *-* | *-* |
| 2p24.3 | case | 13,575,766 | 13,628,120 | 52,355 | Gain | *-* | *-* |
| 2q14.1 | case | 115,523,174 | 115,536,791 | 13,618 | Loss | ***DPP10*** | *-* |
| 2q32.1 | case | 186,479,602 | 186,997,541 | 517,940 | Gain | *FSIP2* | *FSIP2* |
| 2q32.1 | case | 187,880,937 | 187,950,140 | 69,204 | Loss | *-* | *-* |
| 2q36.1 | case | 224,562,229 | 224,576,402 | 14,174 | Loss | *-* | *-* |
| 2q36.3 | case | 228,516,984 | 228,521,978 | 4,995 | Loss | *-* | *-* |
| 2q37.3 | case | 241,506,669 | 241,525,691 | 19,023 | Gain | *RNPEPL1* | *RNPEPL1* |
| 2q37.3 | case | 242,031,107 | 242,036,769 | 5,663 | Gain | *SNED1,MTERFD2* | *MTERFD2* |
| 2q37.3 | case | 242,031,107 | 242,036,769 | 5,663 | Gain | *SNED1,MTERFD2* | *MTERFD2* |
| 3p22.1 | case | 40,003,159 | 40,014,390 | 11,232 | Loss | *MYRIP* | *-* |
| 3p24.1 | case | 29,482,046 | 29,486,572 | 4,527 | Loss | *RBMS3* | *-* |
| 3p25.1 | case | 13,345,450 | 13,367,406 | 21,957 | Gain | *NUP210* | *NUP210* |
| 3p25.1 | case | 13,345,450 | 13,367,406 | 21,957 | Gain | *NUP210* | *NUP210* |
| 3p25.1 | case | 13,357,986 | 13,367,406 | 9,421 | Loss | *NUP210* | *NUP210* |
| 3p26.1 | case | 4,477,911 | 4,531,076 | 53,166 | Loss | ***SUMF1*** | ***SUMF1*** |
| 3q11.2 | case | 94,173,895 | 94,562,501 | 388,607 | Gain | *-* | *-* |
| 3q28 | case | 189,057,350 | 189,069,410 | 12,061 | Gain | *-* | *-* |
| 3q29 | case | 194,238,390 | 194,275,435 | 37,046 | Gain | *-* | *-* |
| 4q21.21 | case | 81,941,456 | 81,954,086 | 12,631 | Loss | *BMP3* | *BMP3* |
| 4q24 | case | 103,578,637 | 103,662,776 | 84,140 | Loss | *MANBA* | *MANBA* |
| 4q32.1 | case | 157,703,801 | 157,797,342 | 93,542 | Gain | *PDGFC* | *PDGFC* |
| 4q35.1 | case | 186,117,162 | 187,061,084 | 943,923 | Loss | *SORBS2,PDLIM3,CCDC110,KIAA1430,TLR3,ANKRD37,C4orf47,SNX25,LRP2BP,UFSP2* | *SORBS2,PDLIM3,CCDC110,TLR3,ANKRD37,C4orf47,SNX25,LRP2BP,UFSP2* |
| 4q35.2 | case | 187,580,706 | 187,605,476 | 24,771 | Gain | *FAT1* | *FAT1* |
| 5p14.1 | case | 28,640,898 | 28,719,681 | 78,784 | Loss | *-* | *-* |
| 5p15.33 | case | 2,782,587 | 2,789,932 | 7,346 | Loss | *-* | *-* |
| 5q13.3 | case | 76,003,258 | 76,029,372 | 26,115 | Gain | ***F2R,IQGAP2*** | ***F2R*** |
| 6p21.31 | case | 35,394,489 | 35,395,546 | 1,058 | Gain | *PPARD* | *-* |
| 6p21.33 | case | 31,016,978 | 31,038,976 | 21,999 | Loss | *-* | *-* |
| 6p21.33 | case | 31,016,978 | 31,038,976 | 21,999 | Loss | *-* | *-* |
| 6p24.3 | case | 7,391,921 | 7,408,678 | 16,758 | Loss | *RIOK1* | *RIOK1* |
| 6q16.1 | case | 95,486,776 | 95,676,106 | 189,331 | Loss | *-* | *-* |
| 6q16.1 | case | 95,946,807 | 95,968,991 | 22,185 | Loss | *-* | *-* |
| 6q21 | case | 109,786,980 | 109,954,252 | 167,273 | Loss | *AK9,ZBTB24* | *AK9,ZBTB24* |
| 7p11.2 | case | 54,750,433 | 54,757,128 | 6,696 | Loss | *-* | *-* |
| 7p11.2 | case | 56,291,170 | 56,357,799 | 66,630 | Gain | *-* | *-* |
| 7p12.3 | case | 48,554,584 | 48,633,056 | 78,473 | Gain | *ABCA13* | *ABCA13* |
| 7p21.2 | case | 14,979,045 | 15,000,359 | 21,315 | Loss | *-* | *-* |
| 7p21.2 | case | 16,351,568 | 16,401,190 | 49,623 | Loss | *ISPD* | *-* |
| 7p21.3 | case | 7,341,918 | 7,344,571 | 2,654 | Loss | *-* | *-* |
| 7p22.3 | case | 44,935 | 83,185 | 38,251 | Gain | *-* | *-* |
| 7q11.21 | case | 61,852,895 | 62,360,358 | 507,464 | Loss | *-* | *-* |
| 7q22.1 | case | 100,153,333 | 100,180,060 | 26,728 | Gain | *LRCH4,AGFG2,SAP25* | *LRCH4,AGFG2,SAP25* |
| 7q31.31 | case | 120,705,246 | 120,816,763 | 111,518 | Loss | *CPED1* | *CPED1* |
| 8p12 | case | 34,060,249 | 34,180,559 | 120,311 | Loss | *-* | *-* |
| 8p22 | case | 14,286,552 | 14,326,637 | 40,086 | Loss | *SGCZ* | *-* |
| 8p22 | case | 15,698,099 | 15,732,889 | 34,791 | Loss | *-* | *-* |
| 8p23.3 | case | 2,142,267 | 2,150,224 | 7,958 | Loss | *-* | *-* |
| 8q12.3 | case | 65,604,777 | 65,628,180 | 23,404 | Gain | *CYP7B1* | *-* |
| 8q24.13 | case | 125,438,835 | 125,479,663 | 40,829 | Gain | *TRMT12* | *TRMT12* |
| 8q24.13 | case | 125,455,570 | 125,479,663 | 24,094 | Gain | *TRMT12* | *TRMT12* |
| 8q24.21 | case | 130,596,548 | 130,601,285 | 4,738 | Loss | *-* | *-* |
| 8q24.3 | case | 143,098,287 | 143,110,435 | 12,149 | Loss | *-* | *-* |
| 9p21.2 | case | 27,232,748 | 27,253,049 | 20,302 | Loss | *-* | *-* |
| 9q33.1 | case | 119,878,362 | 119,887,577 | 9,216 | Loss | *ASTN2* | *-* |
| 9q34.3 | case | 140,301,447 | 140,392,494 | 91,048 | Gain | *NOXA1,NSMF,PNPLA7,EXD3,ENTPD8* | *NOXA1,NSMF,PNPLA7,ENTPD8* |
| 9q34.3 | case | 140,325,962 | 140,392,494 | 66,533 | Gain | *NOXA1,NSMF,PNPLA7,ENTPD8* | *NOXA1,NSMF,PNPLA7,ENTPD8* |
| 10p11.22 | control | 32,834,043 | 32,909,789 | 75,747 | Gain | *CCDC7,C10orf68* | *CCDC7,C10orf68* |
| 10p14 | control | 6,677,767 | 6,835,673 | 157,907 | Gain | *-* | *-* |
| 10p14 | control | 6,699,236 | 6,831,860 | 132,625 | Gain | *-* | *-* |
| 10p14 | control | 10,601,077 | 10,619,227 | 18,151 | Loss | *-* | *-* |
| 10p15.3 | control | 2,338,198 | 2,408,852 | 70,655 | Gain | *-* | *-* |
| 10q21.2 | control | 63,004,505 | 63,035,242 | 30,738 | Loss | *-* | *-* |
| 10q23.33 | control | 96,566,889 | 96,576,190 | 9,302 | Loss | *CYP2C19* | *-* |
| 11p13 | control | 35,244,058 | 35,257,316 | 13,259 | Gain | *CD44* | *CD44* |
| 11p13 | control | 35,244,058 | 35,257,316 | 13,259 | Gain | *CD44* | *CD44* |
| 11p14.3 | control | 22,960,387 | 24,384,375 | 1,423,989 | Loss | *-* | *-* |
| 11p14.3 | control | 24,073,794 | 24,577,558 | 503,765 | Gain | *LUZP2* | *LUZP2* |
| 11p15.4 | control | 5,137,846 | 5,251,478 | 113,633 | Loss | *OR51V1,OR52A1,OR52A5,HBB* | *OR51V1,OR52A1,OR52A5,HBB* |
| 11p15.4 | control | 6,848,913 | 6,856,817 | 7,905 | Loss | *-* | *-* |
| 11q14.1 | control | 83,556,067 | 83,592,397 | 36,331 | Gain | *DLG2* | *DLG2* |
| 11q21 | control | 92,834,198 | 92,864,441 | 30,244 | Gain | *-* | *-* |
| 12p12.3 | control | 16,476,471 | 16,529,269 | 52,799 | Loss | *MGST1* | *MGST1* |
| 12q21.31 | control | 84,073,580 | 84,100,208 | 26,629 | Gain | *-* | *-* |
| 12q24.32 | control | 126,949,886 | 127,109,451 | 159,566 | Loss | *-* | *-* |
| 12q24.33 | control | 131,433,900 | 131,458,250 | 24,351 | Loss | *GPR133* | *GPR133* |
| 13q12.13 | control | 27,323,607 | 27,348,477 | 24,871 | Loss | *GPR12* | *GPR12* |
| 13q31.1 | control | 83,579,619 | 83,593,140 | 13,522 | Loss | *-* | *-* |
| 14q12 | control | 27,160,411 | 27,185,823 | 25,413 | Loss | *-* | *-* |
| 14q13.1 | control | 33,331,841 | 33,345,632 | 13,792 | Gain | *-* | *-* |
| 14q13.1 | control | 33,651,895 | 33,676,728 | 24,834 | Loss | *NPAS3* | *-* |
| 14q13.3 | control | 37,346,623 | 37,349,468 | 2,846 | Loss | *SLC25A21* | *-* |
| 14q31.2 | control | 83,696,060 | 83,930,466 | 234,407 | Gain | *-* | *-* |
| 14q31.3 | control | 86,898,200 | 86,978,489 | 80,290 | Loss | *-* | *-* |
| 15q21.1 | control | 47,141,066 | 47,156,634 | 15,569 | Loss | *-* | *-* |
| 15q21.2 | control | 52,496,933 | 52,528,193 | 31,261 | Loss | *MYO5C* | *MYO5C* |
| 15q21.3 | control | 54,898,587 | 54,912,500 | 13,914 | Loss | *UNC13C* | *-* |
| 15q22.2 | control | 59,655,578 | 59,721,135 | 65,558 | Gain | *MYO1E* | *MYO1E* |
| 15q26.1 | control | 93,691,158 | 93,704,049 | 12,892 | Gain | *-* | *-* |
| 15q26.3 | control | 99,127,913 | 99,131,822 | 3,910 | Gain | *-* | *-* |
| 16q23.1 | control | 75,985,226 | 76,011,177 | 25,952 | Loss | *-* | *-* |
| 16q23.1 | control | 76,401,869 | 76,423,281 | 21,413 | Gain | *CNTNAP4* | *-* |
| 16q23.1 | control | 77,963,488 | 77,988,934 | 25,447 | Loss | *VAT1L* | *-* |
| 16q24.1 | control | 84,332,600 | 84,336,577 | 3,978 | Loss | *WFDC1* | *-* |
| 16q24.3 | control | 89,162,252 | 89,185,019 | 22,768 | Gain | *ACSF3* | *ACSF3* |
| 17p11.2 | control | 19,952,451 | 19,989,218 | 36,768 | Loss | *SPECC1* | *-* |
| 17p12 | control | 14,101,029 | 14,739,547 | 638,519 | Gain | *COX10,CDRT15,HS3ST3B1* | *COX10,CDRT15,HS3ST3B1* |
| 17p12 | control | 14,749,806 | 15,471,179 | 721,374 | Gain | *CDRT4,TVP23C-CDRT4,PMP22,TVP23C,TEKT3* | *TVP23C-CDRT4,PMP22,TEKT3,CDRT4,TVP23C,TVP23C,TVP23C-CDRT4* |
| 17q12 | control | 32,342,541 | 32,356,013 | 13,473 | Loss | *ASIC2* | *-* |
| 17q21.2 | control | 39,304,357 | 39,314,989 | 10,633 | Loss | *KRTAP4-5* | *KRTAP4-5* |
| 17q24.2 | control | 66,632,565 | 66,642,694 | 10,130 | Gain | *-* | *-* |
| 18p11.21 | control | 11,989,373 | 12,141,372 | 152,000 | Gain | *ANKRD62,IMPA2* | *ANKRD62,IMPA2* |
| 18p11.23,18p11.31 | control | 7,080,135 | 7,578,782 | 498,648 | Gain | *LRRC30,LAMA1,PTPRM* | *LRRC30,LAMA1,PTPRM* |
| 18q12.2 | control | 34,946,168 | 35,078,859 | 132,692 | Gain | *CELF4* | *CELF4* |
| 18q12.2,  18q12.3 | control | 37,089,615 | 37,216,115 | 126,501 | Loss | *-* | *-* |
| 18q21.32 | control | 56,787,566 | 56,902,134 | 114,569 | Gain | *SEC11C,GRP* | *SEC11C,GRP* |
| 18q22.3 | control | 72,031,142 | 72,037,334 | 6,193 | Gain | *-* | *-* |
| 18q22.3 | control | 72,031,142 | 72,041,274 | 10,133 | Gain | *-* | *-* |
| 19q11 | control | 28,485,235 | 28,556,910 | 71,676 | Loss | *-* | *-* |
| 19q11 | control | 28,485,235 | 28,569,401 | 84,167 | Loss | *-* | *-* |
| 19q13.32 | control | 46,719,646 | 46,735,006 | 15,361 | Gain | *IGFL1* | *IGFL1* |
| 1p13.3 | control | 108,185,309 | 108,190,581 | 5,273 | Gain | *VAV3* | *VAV3* |
| 1p13.3 | control | 108,596,968 | 108,609,194 | 12,227 | Loss | *-* | *-* |
| 1p13.3 | control | 108,596,968 | 108,609,194 | 12,227 | Loss | *-* | *-* |
| 1p21.1 | control | 106,885,146 | 106,953,168 | 68,023 | Gain | *-* | *-* |
| 1p33 | control | 46,967,890 | 47,035,643 | 67,754 | Loss | *KNCN,MKNK1,DMBX1* | *KNCN,MKNK1,DMBX1* |
| 1p35.2 | control | 30,348,274 | 30,371,100 | 22,827 | Loss | *-* | *-* |
| 1p36.13 | control | 16,446,349 | 16,479,181 | 32,833 | Loss | *EPHA2* | *EPHA2* |
| 1p36.22 | control | 11,048,188 | 11,063,043 | 14,856 | Gain | *-* | *-* |
| 1p36.32 | control | 3,680,643 | 3,786,245 | 105,603 | Gain | *SMIM1,DFFB,LRRC47,CCDC27,CEP104* | *SMIM1,DFFB,LRRC47,CCDC27,CEP104* |
| 1q24.1 | control | 166,415,259 | 166,460,696 | 45,438 | Loss | *-* | *-* |
| 1q31.1 | control | 186,377,439 | 186,447,591 | 70,153 | Gain | *C1orf27,PDC* | *C1orf27,PDC* |
| 1q44 | control | 246,409,434 | 246,429,058 | 19,625 | Loss | *SMYD3* | *-* |
| 20p13 | control | 586,955 | 600,964 | 14,010 | Loss | *TCF15* | *TCF15* |
| 20q11.21 | control | 29,804,293 | 30,063,342 | 259,050 | Gain | *DEFB116,DEFB115,REM1,DEFB118, DEFB119,DEFB124,DEFB123,DEFB121* | *DEFB116,DEFB115,DEFB118,DEFB119,DEFB124,DEFB123,DEFB121* |
| 20q11.21 | control | 29,889,768 | 29,913,233 | 23,466 | Loss | *DEFB116* | *DEFB116* |
| 20q13.13 | control | 47,411,456 | 47,536,334 | 124,879 | Gain | *PREX1* | *PREX1* |
| 20q13.2 | control | 50,631,802 | 50,635,427 | 3,626 | Gain | *-* | *-* |
| 20q13.32 | control | 57,165,395 | 57,171,596 | 6,202 | Loss | *-* | *-* |
| 21q21.1 | control | 18,720,293 | 18,723,671 | 3,379 | Loss | *-* | *-* |
| 21q21.1 | control | 18,720,293 | 18,723,671 | 3,379 | Loss | *-* | *-* |
| 21q21.1 | control | 18,720,293 | 18,723,671 | 3,379 | Loss | *-* | *-* |
| 21q21.2 | control | 24,819,148 | 24,832,028 | 12,881 | Loss | *-* | *-* |
| 21q21.2 | control | 24,819,148 | 24,832,028 | 12,881 | Loss | *-* | *-* |
| 21q21.3 | control | 29,464,782 | 29,479,764 | 14,983 | Loss | *-* | *-* |
| 21q22.3 | control | 43,882,808 | 43,892,699 | 9,892 | Gain | *RSPH1* | *-* |
| 22q11.21 | control | 18,262,767 | 18,270,779 | 8,013 | Loss | *MICAL3* | *-* |
| 2p13.2 | control | 71,589,618 | 71,658,541 | 68,924 | Loss | *ZNF638* | *ZNF638* |
| 2p13.2 | control | 71,710,068 | 71,724,396 | 14,329 | Gain | *DYSF* | *-* |
| 2p16.2 | control | 52,915,347 | 53,024,050 | 108,704 | Loss | *-* | *-* |
| 2p16.3 | control | 50,889,959 | 50,994,255 | 104,297 | Loss | *NRXN1* | *-* |
| 2p16.3 | control | 52,338,106 | 52,346,715 | 8,610 | Loss | *-* | *-* |
| 2p16.3 | control | 52,690,141 | 52,722,668 | 32,528 | Loss | *-* | *-* |
| 2q12.2,  2q12.1 | control | 105,795,508 | 106,149,270 | 353,763 | Gain | *FHL2,GPR45,C2orf49,TGFBRAP1* | *FHL2,GPR45,C2orf49,TGFBRAP1* |
| 2q14.1 | control | 115,523,174 | 115,530,841 | 7,668 | Loss | *DPP10* | *-* |
| 2q14.1 | control | 115,523,174 | 115,530,841 | 7,668 | Loss | *DPP10* | *-* |
| 2q14.1 | control | 115,523,174 | 115,530,841 | 7,668 | Loss | *DPP10* | *-* |
| 2q14.1 | control | 115,523,174 | 115,530,841 | 7,668 | Loss | *DPP10* | *-* |
| 2q14.1 | control | 115,523,174 | 115,530,841 | 7,668 | Loss | *DPP10* | *-* |
| 2q14.3 | control | 126,070,876 | 126,154,088 | 83,213 | Gain | *-* | *-* |
| 2q22.3 | control | 146,800,596 | 146,885,474 | 84,879 | Loss | *-* | *-* |
| 2q23.3 | control | 154,058,372 | 154,067,245 | 8,874 | Loss | *-* | *-* |
| 2q31.2 | control | 179,853,545 | 179,886,343 | 32,799 | Loss | *CCDC141* | *-* |
| 2q36.1 | control | 222,065,248 | 222,074,201 | 8,954 | Loss | *-* | *-* |
| 2q36.1 | control | 222,065,248 | 222,074,201 | 8,954 | Loss | *-* | *-* |
| 2q36.1 | control | 223,674,322 | 223,700,432 | 26,111 | Loss | *-* | *-* |
| 2q36.1 | control | 224,439,486 | 224,469,380 | 29,895 | Loss | *SCG2* | *SCG2* |
| 2q36.1 | control | 224,555,134 | 224,576,402 | 21,269 | Loss | *-* | *-* |
| 2q36.1 | control | 224,562,229 | 224,576,402 | 14,174 | Loss | *-* | *-* |
| 2q37.3 | control | 242,028,741 | 242,036,769 | 8,029 | Gain | *SNED1,MTERFD2* | *MTERFD2* |
| 2q37.3 | control | 242,031,107 | 242,036,769 | 5,663 | Gain | *SNED1,MTERFD2* | *MTERFD2* |
| 2q37.3 | control | 242,137,702 | 242,164,562 | 26,861 | Loss | *ANO7* | *ANO7* |
| 3p14.2 | control | 60,350,709 | 60,506,349 | 155,641 | Loss | *FHIT* | *-* |
| 3p14.3 | control | 57,031,469 | 57,055,271 | 23,803 | Loss | *ARHGEF3* | *-* |
| 3p21.1 | control | 53,332,363 | 53,457,819 | 125,457 | Gain | *DCP1A* | *DCP1A* |
| 3p24.1 | control | 27,475,351 | 27,652,901 | 177,551 | Gain | *SLC4A7* | *SLC4A7* |
| 3p25.1 | control | 13,345,450 | 13,367,406 | 21,957 | Gain | *NUP210* | *NUP210* |
| 3p25.1 | control | 13,345,450 | 13,367,406 | 21,957 | Gain | *NUP210* | *NUP210* |
| 3p26.3 | control | 455,241 | 504,756 | 49,516 | Loss | *-* | *-* |
| 3q13.11 | control | 103,079,255 | 103,094,740 | 15,486 | Loss | *-* | *-* |
| 3q13.13 | control | 110,788,941 | 110,860,188 | 71,248 | Gain | *PVRL3* | *PVRL3* |
| 3q13.31 | control | 116,595,111 | 117,049,817 | 454,707 | Loss | *-* | *-* |
| 3q26.1 | control | 161,087,857 | 161,226,185 | 138,329 | Gain | *OTOL1,SPTSSB* | *OTOL1* |
| 3q26.31 | control | 174,895,840 | 175,064,809 | 168,970 | Loss | *NAALADL2* | *NAALADL2* |
| 3q26.32 | control | 176,680,084 | 176,824,755 | 144,672 | Gain | *TBL1XR1* | *TBL1XR1* |
| 3q26.33 | control | 179,252,263 | 179,331,178 | 78,916 | Gain | *NDUFB5,MRPL47,ACTL6A* | *NDUFB5,MRPL47,ACTL6A* |
| 3q28 | control | 191,272,414 | 191,291,423 | 19,010 | Loss | *-* | *-* |
| 4p16.1 | control | 9,804,556 | 9,896,760 | 92,205 | Gain | *SLC2A9* | *SLC2A9* |
| 4q21.21 | control | 81,941,456 | 81,950,655 | 9,200 | Loss | *-* | *-* |
| 4q21.21 | control | 81,941,456 | 81,950,655 | 9,200 | Loss | *-* | *-* |
| 4q21.21 | control | 81,941,456 | 81,950,655 | 9,200 | Loss | *-* | *-* |
| 4q26 | control | 118,339,156 | 118,348,459 | 9,304 | Loss | *-* | *-* |
| 4q28.2 | control | 130,469,043 | 130,508,349 | 39,307 | Gain | *-* | *-* |
| 4q32.1 | control | 161,255,092 | 161,409,160 | 154,069 | Loss | *-* | *-* |
| 4q32.3 | control | 169,725,605 | 169,740,931 | 15,327 | Loss | *PALLD* | *-* |
| 4q34.3 | control | 177,539,619 | 177,587,894 | 48,276 | Loss | *-* | *-* |
| 4q35.1 | control | 184,501,675 | 184,577,348 | 75,674 | Gain | *RWDD4* | *RWDD4* |
| 4q35.1 | control | 184,974,967 | 184,995,011 | 20,045 | Gain | *-* | *-* |
| 5p14.1 | control | 28,640,898 | 28,719,681 | 78,784 | Loss | *-* | *-* |
| 5q11.2 | control | 53,299,435 | 53,815,560 | 516,126 | Gain | *ARL15,HSPB3,SNX18* | *ARL15,HSPB3,SNX18* |
| 5q11.2 | control | 57,378,468 | 57,413,380 | 34,913 | Loss | *-* | *-* |
| 5q14.1 | control | 80,459,446 | 80,467,798 | 8,353 | Loss | *RASGRF2* | *-* |
| 5q14.1 | control | 80,459,446 | 80,467,798 | 8,353 | Loss | *RASGRF2* | *-* |
| 5q21.1 | control | 99,248,759 | 99,260,992 | 12,234 | Loss | *-* | *-* |
| 5q23.1 | control | 116,388,731 | 116,414,003 | 25,273 | Loss | *-* | *-* |
| 5q23.2 | control | 123,851,734 | 123,886,769 | 35,036 | Gain | *-* | *-* |
| 5q23.2 | control | 123,853,534 | 123,886,769 | 33,236 | Gain | *-* | *-* |
| 5q31.2 | control | 138,084,269 | 138,209,404 | 125,136 | Gain | *CTNNA1,LRRTM2* | *CTNNA1,LRRTM2* |
| 5q34 | control | 166,221,489 | 166,252,148 | 30,660 | Loss | *-* | *-* |
| 5q35.2 | control | 176,081,907 | 176,085,391 | 3,485 | Gain | *TSPAN17* | *TSPAN17* |
| 6p12.3 | control | 51,460,207 | 51,480,486 | 20,280 | Loss | *PKHD1* | *-* |
| 6p21.2 | control | 40,066,652 | 40,085,245 | 18,594 | Loss | *-* | *-* |
| 6p21.2 | control | 40,066,652 | 40,096,889 | 30,238 | Loss | *-* | *-* |
| 6p21.33 | control | 30,608,636 | 30,624,880 | 16,245 | Gain | *C6orf136,ATAT1,DHX16* | *C6orf136,ATAT1,DHX16* |
| 6p21.33 | control | 31,016,978 | 31,038,976 | 21,999 | Loss | *-* | *-* |
| 6p22.1 | control | 27,998,258 | 28,018,944 | 20,687 | Gain | *-* | *-* |
| 6p22.1 | control | 29,644,502 | 29,656,578 | 12,077 | Loss | *ZFP57* | *ZFP57* |
| 6q14.1 | control | 77,419,099 | 77,502,291 | 83,193 | Gain | *-* | *-* |
| 6q22.31 | control | 125,522,271 | 125,587,961 | 65,691 | Gain | *TPD52L1* | *TPD52L1* |
| 6q25.2 | control | 153,833,568 | 153,902,816 | 69,249 | Gain | *-* | *-* |
| 6q27 | control | 165,317,841 | 165,343,557 | 25,717 | Gain | *-* | *-* |
| 6q27 | control | 169,248,186 | 169,265,795 | 17,610 | Loss | *-* | *-* |
| 7p14.1 | control | 39,833,350 | 39,942,647 | 109,298 | Loss | *-* | *-* |
| 7p21.2 | control | 16,058,837 | 16,062,239 | 3,403 | Loss | *-* | *-* |
| 7p21.3 | control | 8,827,959 | 8,856,074 | 28,116 | Loss | *-* | *-* |
| 7p21.3 | control | 12,185,607 | 12,218,360 | 32,754 | Loss | *-* | *-* |
| 7p21.3 | control | 13,492,428 | 13,502,530 | 10,103 | Loss | *-* | *-* |
| 7p22.1 | control | 4,880,954 | 4,909,983 | 29,030 | Gain | *PAPOLB,RADIL* | *PAPOLB* |
| 7p22.1 | control | 4,962,688 | 4,975,956 | 13,269 | Gain | *MMD2* | *MMD2* |
| 7p22.1,  7p22.2 | control | 4,474,132 | 4,667,201 | 193,070 | Gain | *-* | *-* |
| 7p22.2 | control | 3,101,279 | 3,126,241 | 24,963 | Gain | *-* | *-* |
| 7p22.2 | control | 3,445,311 | 3,455,249 | 9,939 | Gain | *SDK1* | *-* |
| 7p22.2 | control | 3,710,751 | 3,765,004 | 54,254 | Loss | *SDK1* | *-* |
| 7p22.2 | control | 4,310,365 | 4,381,743 | 71,379 | Loss | *-* | *-* |
| 7p22.3 | control | 936,765 | 1,024,581 | 87,817 | Gain | *ADAP1,COX19,CYP2W1* | *ADAP1,COX19,CYP2W1* |
| 7p22.3 | control | 1,022,728 | 1,039,457 | 16,730 | Loss | *CYP2W1,C7orf50* | *CYP2W1,C7orf50* |
| 7q11.21 | control | 62,043,624 | 62,699,114 | 655,491 | Gain | *-* | *-* |
| 7q11.21 | control | 64,679,561 | 65,088,807 | 409,247 | Loss | *ZNF92* | *ZNF92* |
| 7q11.21 | control | 65,420,340 | 65,521,227 | 100,888 | Gain | *GUSB* | *GUSB* |
| 7q21.11 | control | 78,092,244 | 78,094,640 | 2,397 | Loss | *MAGI2* | *-* |
| 7q31.1 | control | 111,265,262 | 111,362,942 | 97,681 | Loss | *-* | *-* |
| 7q31.2 | control | 114,778,640 | 114,900,684 | 122,045 | Loss | *-* | *-* |
| 7q34 | control | 138,363,896 | 138,376,184 | 12,289 | Loss | *-* | *-* |
| 8p21.3 | control | 19,109,719 | 19,115,604 | 5,886 | Loss | *-* | *-* |
| 8p21.3 | control | 20,078,643 | 20,134,377 | 55,735 | Gain | *ATP6V1B2,LZTS1* | *LZTS1* |
| 8p21.3 | control | 20,150,536 | 20,280,942 | 130,407 | Gain | *-* | *-* |
| 8p21.3 | control | 20,315,601 | 20,367,070 | 51,470 | Gain | *-* | *-* |
| 8p23.2 | control | 2,731,382 | 2,748,965 | 17,584 | Loss | *-* | *-* |
| 8q11.23 | control | 53,343,969 | 53,455,537 | 111,569 | Gain | *FAM150A* | *FAM150A* |
| 8q12.1 | control | 59,788,913 | 59,796,616 | 7,704 | Loss | *TOX* | *-* |
| 8q21.13 | control | 80,251,214 | 80,274,461 | 23,248 | Loss | *-* | *-* |
| 8q24.13 | control | 125,407,426 | 125,479,663 | 72,238 | Gain | *TRMT12* | *TRMT12* |
| 8q24.13 | control | 125,438,835 | 125,479,663 | 40,829 | Gain | *TRMT12* | *TRMT12* |
| 8q24.13 | control | 125,438,835 | 125,485,201 | 46,367 | Gain | *TRMT12* | *TRMT12* |
| 8q24.21 | control | 129,218,047 | 129,224,053 | 6,007 | Loss | *-* | *-* |
| 9p21.2 | control | 25,627,885 | 25,658,472 | 30,588 | Loss | *-* | *-* |
| 9p21.3 | control | 22,724,188 | 22,819,064 | 94,877 | Loss | *-* | *-* |
| 9q31.3 | control | 113,431,606 | 113,552,952 | 121,347 | Gain | *MUSK* | *MUSK* |
| 9q33.3 | control | 128,926,091 | 128,966,109 | 40,019 | Loss | *-* | *-* |
