# Supplementary material for: Rare Copy Number Variants Identified Suggest the Regulating Pathways in Hypertension-Related Left Ventricular Hypertrophy
Source: PLoS One. 2016 Mar 1;11(3):e0148755. doi: 10.1371/journal.pone.0148755 (PMC4773219; doi:10.1371/journal.pone.0148755)
Supplement: S7 Table — (DOC) [file pone.0148755.s007.doc]

**S7 Table. Gene ontology and pathway analyses identified in the hypertension-related LVH from the replication study**

| **Term** | **P Value** | **Genes** |
| --- | --- | --- |
| ***DAVID:*** |  |  |
| IPR006210:EGF-like | 0.010 | *NRG3, ATRNL1, SNED1, FAT1, FAT2, FAM5C, MEGF11* |
| GO:0043167~ion binding | 0.040 | *PXDN, PPARD, CNDP1, CNDP2, RNPEPL1, TRPV3, DUOX1, PDLIM3, ZNF880, KCNIP4, MUTYH, MCTP2, ZNF300, ZNF407, GUCY1A2, ZNF506, ZSWIM6, CYB5A, PADI4, ZBTB24, ZSWIM5, TRIM37, MYRIP, ATP9B, TESK2, ZNF480, ABLIM1, AGFG2, ZNF610, ITGAE, ZBTB11, BRSK2, MYO9A, TOE1, MMACHC, SORBS2, FAT1, FAT2, ENTPD8, CERK, STK38L, USP32, ZNF528, SPHK1, GABRA5, GSG2, MANBA, CYP7B1, CDH15, SNED1, FLG, RPS6KA2, SUMF1* |
| GO:0006800~oxygen and reactive oxygen species metabolic process | 0.026 | *PXDN, NOXA1, DUOX1, PRDX1* |
| ***IPA:*** |  |  |
| Infectious diseases | 4.72E-02 – 7.39E-07 | *CD46, IFITM1, IFITM2, IFITM5, F2R, TLR3, IFNAR2* |
| Respiratory diseases | 7.39E-07 - 4.06E-02 | *CD46, IFITM1, IFITM2, IFITM5, NSMF, PTGER3, CYP7B1, AT1, FUT8, MEGF11, TLR3, NPAS3* |
| Molecular transport | 1.39E-03 - 3.77E-02 | *PRKG1, PTGER3, F2R, FAT1, PPARD, TLR3* |
| Cell death and survival | 1.73E-03 - 4.72E-02 | *FAT1, PPARD, CD46, TLR3, PRKG1* |
| Energy production | 1.73E-03 - 1.71E-02 | *F2R, PRKG1, PPARD* |
| Cardiovascular development and function | 7.24E-04 - 4.17E-02 | *F2R, PDGFC, TLR3, PRKG1, PTGER3* |
| Organismal development | 7.48E-04 - 4.77E-02 | *F2R, PDGFC, TLR3, PPARD, MANBA, OAT, NPAS3, BMP3, PTGER3, IFITM5, IQGAP2, FAT1, FUT8, PARD3, PRKG1, SNX27, PDGFC, BMP3* |
