# Supplementary material for: Rare Copy Number Variants Identified Suggest the Regulating Pathways in Hypertension-Related Left Ventricular Hypertrophy
Source: PLoS One. 2016 Mar 1;11(3):e0148755. doi: 10.1371/journal.pone.0148755 (PMC4773219; doi:10.1371/journal.pone.0148755)
Supplement: S8 Table — (DOC) [file pone.0148755.s008.doc]

**S8 Table. Top 5 networks identified by Ingenuity (IPA) from the replication study**

| **Top diseases & functions** | **Molecules in network** |
| --- | --- |
| Infectious diseases, respiratory disease, cardiovascular system development and function | *NOXA1, NSMF, PDGFC, PPARD, PRKG1, PTGER3, SNX27, TLR3* |
| Cell cycle, DNA replication, recombination and repair, developmental disorder | *OAT, PPFIBP1, SNX25, SUMF1, TRUB1, TUFT1, UFSP2* |
| Carbohydrate metabolism, lipid metabolism, small molecules biochemistry | *NPAS3, PDLIM3, PNPLA7, RMBS3, RIOK1, SHF, STK38L, ZBTB4* |
| Connective tissue disorder, developmental disorder, hereditary disorder | *PARD3, RNPELPL1, SGCZ, SMCO2, ZNF506* |
| Gene expression, cell death and survival, cell morphology | *SAP25, SNED1, SORBS2* |
