# Supplementary material for: Rare Copy Number Variants Identified Suggest the Regulating Pathways in Hypertension-Related Left Ventricular Hypertrophy
Source: PLoS One. 2016 Mar 1;11(3):e0148755. doi: 10.1371/journal.pone.0148755 (PMC4773219; doi:10.1371/journal.pone.0148755)
Supplement: S9 Table — Gene ontology and pathway analyses identified using DAVID in the hypertension patients without LVH (denoted as controls) from (i) Illumina 660W; (ii) Illumina Omni Express; (iii) combination from Illumina 660W and Illumina Omni Express datasets. (DOC) [file pone.0148755.s009.doc]

**S9 Table. Gene ontology and pathway analyses identified using DAVID in the hypertension patients without LVH (denoted as controls) from (i) Illumina 660W; (ii) Illumina Omni Express; (iii) combination from Illumina 660W and Illumina Omni Express datasets.**

(i)

| **Term** | **P Value** | **Genes** | **FDR** |
| --- | --- | --- | --- |
|  |  |  |  |
| GO:0006796~phosphate metabolic process | 0.0317 | *FCER1A, NDUFA3, ERBB4, TRIM28, ABI2, STRADB, CDC25A, GALK2, MTMR3, PKN3, ADRA2C, ABL1, CDK20* | 39.881 |
| GO:0006793~phosphorus metabolic process | 0.0317 | *FCER1A, NDUFA3, ERBB4, TRIM28, ABI2, STRADB, CDC25A, GALK2, MTMR3, PKN3, ADRA2C, ABL1, CDK20* | 39.881 |
| GO:0016310~phosphorylation | 0.0448 | *FCER1A, GALK2, NDUFA3, ERBB4, PKN3, TRIM28, ABI2, ADRA2C, STRADB, ABL1, CDK20* | 51.504 |
| GO:0043549~regulation of kinase activity | 0.0347 | *FCER1A, VAV3, ADCY8, NF1, ADRA2C, STRADB, CDC25A* | 42.743 |
| GO:0051338~regulation of transferase activity | 0.0411 | *FCER1A, VAV3, ADCY8, NF1, ADRA2C, STRADB, CDC25A* | 48.443 |
| GO:0009165~nucleotide biosynthetic process | 0.0380 | *ITGB1BP3, ATP10B, ADCY8, ATP11A, NME6* | 45.710 |
| GO:0034654~nucleobase, nucleoside, nucleotide and nucleic acid biosynthetic process | 0.0425 | *ITGB1BP3, ATP10B, ADCY8, ATP11A, NME6* | 49.641 |
| GO:0034404~nucleobase, nucleoside and nucleotide biosynthetic process | 0.0425 | *ITGB1BP3, ATP10B, ADCY8, ATP11A, NME6* | 49.641 |
| GO:0005096~GTPase activator activity | 0.0208 | *VAV3, PREX2, NF1, TBC1D13, ASAP1, ARHGAP15* | 24.048 |

**(ii)**

| **Term** | **P Value** | **Genes** | **FDR** |
| --- | --- | --- | --- |
|  |  |  |  |
| GO:0050839~cell adhesion molecule binding | 0.0095 | *PTPRM, PVRL3, CTNNA1* | 11.458 |
| hsa04520:Adherens junction | 0.0543 | *PTPRM, PVRL3, CTNNA1* | 39.969 |
| GO:0005912~adherens junction | 0.0550 | *PTPRM, PVRL3, FHL2, CTNNA1* | 49.813 |
| IPR001331:Guanine-nucleotide dissociation stimulator, CDC24, conserved site | 0.0402 | *ARHGEF3, RASGRF2, PREX1* | 41.251 |
| IPR000219:Dbl homology (DH) domain | 0.0474 | *ARHGEF3, RASGRF2, PREX1* | 46.692 |

(iii)

| **Term** | **P Value** | **Genes** | **FDR** |
| --- | --- | --- | --- |
| GO:0009165~nucleotide biosynthetic process | 0.0156 | *ITGB1BP3, ATP10B, ADCY8, ATP9B, GUCY1A2, ATP11A, ATP6V1B2, NME6* | 22.708 |
| GO:0006164~purine nucleotide biosynthetic process | 0.0177 | *ATP10B, ADCY8, ATP9B, GUCY1A2, ATP11A, ATP6V1B2, NME6* | 25.301 |
| GO:0034654~nucleobase, nucleoside, nucleotide and nucleic acid biosynthetic process | 0.0188 | *ITGB1BP3, ATP10B, ADCY8, ATP9B, GUCY1A2, ATP11A, ATP6V1B2, NME6* | 26.695 |
| GO:0034404~nucleobase, nucleoside and nucleotide biosynthetic process | 0.0188 | *ITGB1BP3, ATP10B, ADCY8, ATP9B, GUCY1A2, ATP11A, ATP6V1B2, NME6* | 26.695 |
| GO:0044271~nitrogen compound biosynthetic process | 0.0387 | *ITGB1BP3, ATP10B, COX10, ADCY8, ATP9B, GUCY1A2, SULT1A2, ATP11A, ATP6V1B2, NME6* | 47.563 |
| GO:0006163~purine nucleotide metabolic process | 0.0465 | *ATP10B, ADCY8, ATP9B, GUCY1A2, ATP11A, ATP6V1B2, NME6* | 54.032 |
| IPR001331:Guanine-nucleotide dissociation stimulator, CDC24, conserved site | 0.0105 | *ARHGEF3, VAV3, RASGRF2, PREX1, PREX2* | 14.061 |
| IPR000219:Dbl homology (DH) domain | 0.0143 | *ARHGEF3, VAV3, RASGRF2, PREX1, PREX2* | 18.696 |
| GO:0005089~Rho guanyl-nucleotide exchange factor activity | 0.0173 | *ARHGEF3, VAV3, RASGRF2, PREX1, PREX2* | 21.512 |
| GO:0005088~Ras guanyl-nucleotide exchange factor activity | 0.0305 | *ARHGEF3, VAV3, RASGRF2, PREX1, PREX2* | 34.851 |
| GO:0035023~regulation of Rho protein signal transduction | 0.0499 | *ARHGEF3, VAV3, RASGRF2, PREX1, PREX2* | 56.688 |
| GO:0006793~phosphorus metabolic process | 0.0171 | *FCER1A, IMPA2, NDUFB5, NDUFA3, PTPRM, ERBB4, COX10, PTPRN2, TRIM28, BRSK2, ABI2, MKNK1, STRADB, ATP6V1B2, EPHA2, CDC25A, GALK2, MTMR3, MUSK, PKN3, ADRA2C, ABL1, CDK20* | 24.577 |
| GO:0006796~phosphate metabolic process | 0.0171 | *FCER1A, IMPA2, NDUFB5, NDUFA3, PTPRM, ERBB4, COX10, PTPRN2, TRIM28, BRSK2, ABI2, MKNK1, STRADB, ATP6V1B2, EPHA2, CDC25A, GALK2, MTMR3, MUSK, PKN3, ADRA2C, ABL1, CDK20* | 24.577 |
| GO:0046578~regulation of Ras protein signal transduction | 0.0093 | *ARHGEF3, VAV3, RASGRF2, PREX1, PREX2, NF1, TBC1D13, ASAP1, ADAP1* | 14.192 |
| GO:0051056~regulation of small GTPase mediated signal transduction | 0.0253 | *ARHGEF3, VAV3, RASGRF2, PREX1, PREX2, NF1, TBC1D13, ASAP1, ADAP1* | 34.230 |
| GO:0005083~small GTPase regulator activity | 0.0314 | *ARHGEF3, VAV3, RASGRF2, PREX1, PREX2, NF1, TBC1D13, ASAP1, ADAP1* | 35.695 |
| GO:0030695~GTPase regulator activity | 0.0455 | *ARHGEF3, VAV3, RASGRF2, PREX1, PREX2, NF1, TBC1D13, ASAP1, ARHGAP15, DOCK5, ADAP1* | 47.565 |
| GO:0015914~phospholipid transport | 0.0108 | *KCNN4, ATP10B, ATP9B, ATP11A* | 16.204 |
| IPR006539:ATPase, P-type, phospholipid-translocating, flippase | 0.0150 | *ATP10B, ATP9B, ATP11A* | 19.441 |
| GO:0004012~phospholipid-translocating ATPase activity | 0.0167 | *ATP10B, ATP9B, ATP11A* | 20.828 |
| GO:0015247~aminophospholipid transporter activity | 0.0167 | *ATP10B, ATP9B, ATP11A* | 20.828 |
